# Bayesian inference of gene regulatory networks at stochastic steady state

**DOI:** 10.1098/rsif.2026.0040

**Published:** 2026-09-09

**Authors:** Anshi Gupta, Ryeongkyung Yoon, Kresimir Josic

**Affiliations:** ^1^ Mathematics, University of Houston, Houston, TX, USA; ^2^ Biology and Biochemistry, University of Houston, Houston, TX, USA; ^3^ Mathematics and Computer Science, Wabash College, Crawfordsville, IN, USA

**Keywords:** gene regulatory networks, Bayesian inference, chemical Langevin equation, stochastic gene expression, network structure inference

## Abstract

Gene regulatory networks (GRNs) form the regulatory backbone that coordinates gene expression. The architecture of GRNs shapes their function and constrains the biochemical pathways through which information flows. Inferring the structure of regulatory interactions is thus essential for understanding biological systems and designing targeted therapies. Despite substantial progress in GRN inference, most approaches—from statistical methods to deep learning—do not take into account fundamental biochemical processes that drive regulatory dynamics. To address this shortcoming, here, we present a novel Bayesian inference approach based on using the chemical Langevin equation as a model of gene expression dynamics at stochastic equilibrium. Interactions in GRNs are sparse, and we thus use a regularized horseshoe prior enabling selective shrinkage of unsupported interactions while identifying strong regulatory edges. We evaluate our method using synthetic gene expression data, allowing for benchmarking against a known ground truth. Our approach allows us to infer kinetic parameters, identify network structure and infer regulatory cycles without the need to observe transient dynamics. This Bayesian alternative to current methods thus provides both biological interpretability and structural identifiability in GRN inference.

## 1. Introduction

Gene regulatory networks (GRNs) play a central role in controlling gene expression and coordinating cellular functions during development, cell differentiation and responses to environmental stimuli [1]. Inferring the structure of GRNs is thus essential to understanding the regulatory mechanisms that govern complex biological processes, with applications ranging from the characterization of synthetic gene circuits [2,3] and the analysis of single-cell transcriptomic time-series data [4], to the identification of dysregulated regulatory interactions in disease contexts, such as cancer and developmental disorders, where key circuit components represent potential therapeutic targets. Despite extensive efforts to develop experimental and statistical approaches for GRN inference, reconstructing network structure and dynamics from gene expression data remains a challenging inverse problem. Key obstacles include stochasticity in gene expression [5], sparse connectivity between regulatory components [6] and nonlinear interactions within the network [7]. Sparse sampling and measurements near dynamical equilibrium further compound these challenges, making it difficult to resolve dynamic regulatory interactions from gene expression data.

Previous approaches have typically attempted to infer regulatory interactions from high-throughput, high-dimensional biological data, often without relying on detailed mechanistic descriptions. Techniques such as mutual information [8,9], regression-based inference [10,11] and deep learning [12] have been employed to recover GRN structure, aiming to balance predictive performance with interpretability. Although such methods capture complex, nonlinear gene interactions, issues with interpretability and validation persist. On the other hand, many model-based inference methods require measurements of transient dynamics: since multiple parameter combinations are consistent with identical steady-state behaviour, observations of transients are often needed to ensure parameter identifiability [13].

Few existing methods for GRN inference capture stochastic gene expression dynamics, enforce biologically motivated sparsity to infer network structure, interaction types and kinetic parameters from data in stochastic equilibrium, while providing principled uncertainty quantification [14,15]. To address this problem, we introduce a Bayesian inference framework that describes intrinsic stochasticity using the chemical Langevin equation (CLE) [16,17] and incorporates a regularized horseshoe prior [18] to reflect the sparsity of interactions in GRNs. We apply our method to synthetic data from GRNs at stochastic equilibrium, with measurements sampled from the stationary distribution. The proposed inference framework does not require stationarity; the CLE-based likelihood applies equally to transient and stationary data. We focus on the stationary regime because at equilibrium, deterministic methods carry no information about regulatory interactions, making it a more challenging and less studied setting. At equilibrium, the temporal structure of fluctuations provides information about regulatory interactions and kinetic parameters that is not captured by the moments of the distribution alone [19]. Our approach is closely related to the SINDy algorithm [20–22] and is conceptually related to fluctuation–dissipation relations in statistical physics, where equilibrium fluctuations reveal response properties without requiring external perturbations [23]. We show that we can identify the underlying network structure and infer kinetic parameters and interaction types, producing interpretable outputs with well-calibrated uncertainty estimates. Notably, we can recover features such as regulatory cycles and autoregulatory interactions, which are often missed by other Bayesian methods [24].

Our approach is based on a mechanistic model of regulatory interactions, in contrast to non-parametric models, which offer greater flexibility but may sacrifice interpretability and biological grounding. We demonstrate reliable performance on small-scale networks, even when only partially observed. We thus lay the foundation for a robust, mechanistic network inference framework applicable in more complex settings.

## 2. Materials and methods

Our goal is to infer the structure and regulatory parameters of GRNs from stationary gene expression data. To do so, we fit a mechanistic model of gene expression to data using a Bayesian approach. The resulting posterior distributions determine network topology (presence/absence of edges), interaction types (activation or repression) and reaction rates, while providing principled uncertainty quantification for all inferred quantities.

### 2.1. Gene regulatory network model

A GRN is a system in which regulatory molecules (primarily transcription factors (TFs), but also non-coding RNAs and other factors) regulate gene expression through activation or repression. Here, we concentrate on regulation by TFs and represent a GRN as a directed graph with nodes corresponding to genes (or more precisely, gene products). For each gene, i, we denote by $X_{i}(t)$ the abundance of the corresponding TF at time t, and let $X(t)=(X_{1}(t),\ldots ,X_{p}(t))$ be the state of the network, where p is the total number of TFs and genes. We assume reactions in a system at constant volume so that $X_{i}(t)$ can represent the molecular count or concentration. A directed edge, i→j, represents a regulatory interaction where gene i (via its product) impacts the expression of gene j. Edges carry a sign indicating *activation* (the TF increases the target's transcription rate) or *repression* (the TF decreases it). We allow for autoregulation which is represented by a self-loop. At the molecular level, regulation arises when TFs bind promoter or enhancer regions and modulate RNA polymerase recruitment [25,26]. Although other regulatory mechanisms are possible, here, we consider only transcriptional regulation via activation and repression.

The relationship between TF concentrations and transcriptional output is nonlinear, owing to saturation at high concentrations and regulatory thresholds [27,28]. The dependence of transcription rate on TF concentration is commonly described using sigmoidal (Hill-type) functions, which approximate equilibrium TF–promoter binding and reproduce the nonlinear dose–response curves observed experimentally [29–31]. We model the effect of a single regulator on transcription rate by

(2.1) $$f_{a}(X)=\beta \frac{X^{n}}{K^{n}+X^{n}}\;\text{and}\;f_{r}(X)=\gamma \frac{K^{n}}{K^{n}+X^{n}},$$

where β and γ denote the maximal transcription rates under activation ($f_{a}$) and repression ($f_{r}$), respectively. The parameter K is the half-maximal effective concentration, representing the TF level at which transcription reaches half of its maximal rate. The Hill coefficient, n, determines the steepness of the input–output curve and reflects the apparent cooperativity of the regulatory interactions. For simplicity, we assume negligible basal transcription, but our framework can be extended to include it.

Many genes are regulated by multiple TFs, and promoter activity often reflects their combined influence. Because the molecular mechanisms of combinatorial TF regulation are often unknown or intractable, promoter responses are typically modelled using coarse-grained logic functions including AND, OR and hybrid gates [30,32–34]. Here, we focus on the *AND gate* and assume that transcription occurs only when all required TFs are bound to their respective binding sites. This represents a common regulatory motif with multiple inputs converging to activate transcription. While we only consider the case of AND gates with two TFs, the framework can be extended to other logic gates (OR, NOT and hybrid) and to genes with more than two regulators.

We use the following functions to describe AND-gate-type regulation for different combinations of two activators and repressors [35]:

(2.2) $$\begin{array}{rl} & \text{activation-activation (AND logic):}\\ & \;H_{aa}(X_{1},X_{2})=\frac{X_{1}^{n_{1}}X_{2}^{n_{2}}}{\left(X_{1}^{n_{1}}+K_{1}^{n_{1}})(X_{2}^{n_{2}}+K_{2}^{n_{2}}\right)},\\ & \text{repression-repression (NOR logic):}\\ & \;H_{rr}(X_{1},X_{2})=\frac{K_{1}^{n_{1}}K_{2}^{n_{2}}}{\left(X_{1}^{n_{1}}+K_{1}^{n_{1}})(X_{2}^{n_{2}}+K_{2}^{n_{2}}\right)}\;\text{and}\\ & \text{activation-repression (AND logic):}\\ & \;H_{ar}(X_{1},X_{2})=\frac{X_{1}^{n_{1}}K_{2}^{n_{2}}}{\left(X_{1}^{n_{1}}+K_{1}^{n_{1}})(X_{2}^{n_{2}}+K_{2}^{n_{2}}\right)}. \end{array}$$

These normalized functions are multiplied by the maximal transcription rate to obtain the actual production rate.

Gene expression is inherently stochastic at the single-cell level owing to the discrete nature of molecular events and the probabilistic timing of biochemical reactions. *Intrinsic noise* is owing to the probabilistic nature of biochemical events (e.g. TF or RNA polymerase binding), whereas *extrinsic noise* stems from cell-to-cell variability in global factors such as growth state and environmental influences [4,5,36,37]. We focus on intrinsic noise as it directly reflects the biochemical processes we model (transcription, degradation and regulation), as well as the regulatory topology. Incorporating extrinsic noise would require including additional sources of variability.

We validate our approach using synthetic data. To do so, we generate sample expression trajectories using the *stochastic simulation algorithm* (SSA), with the reaction rates as defined in equations (2.1) and (2.2). The SSA produces trajectories whose probability law is specified by the *chemical master equation* (CME), which determines the evolution of the probability distribution over all possible molecular states [17].

Direct inference using the CME quickly becomes computationally intractable. Instead, we use a discretized form (Euler–Maruyama) of the CLE [16,38]. The CLE is a mesoscopic stochastic differential equation (SDE) whose sample paths are distributed approximately according to the probability law described by the CME. Intuitively, the CLE replaces the discrete change in TF number over small time intervals by Gaussian increments with reaction propensities determining the mean and variance of each increment. This diffusion approximation is appropriate under well-stirred conditions and is valid when molecule numbers are sufficiently large so that continuous state variables are appropriate, yet small enough that stochastic fluctuations remain important. Specifically, the CLE provides an accurate approximation to the CME with molecule counts per species at around 100 or above [16,38,39], consistent with experimentally measured TF copy numbers in bacterial cells, which typically range from tens to several thousands of molecules per cell [4]. The approximation breaks down when molecule numbers fall below about 20, as the Gaussian approximation to the discrete reaction counts becomes inaccurate and the diffusion term can produce unphysical negative counts [38,39]. In all examples we present, counts were maintained approximately in the range of 100–400 molecules, well within the mesoscopic regime in which the CLE is valid. The discretized CLE yields tractable transition probabilities, enabling efficient likelihood-based parameter inference [40–42].

### 2.2. Example: single-gene autoregulation

As a first example, and to explain notation, we consider single-gene autoregulation [43,44]. This simplest of GRNs can exhibit rich dynamics and serves as the basis for multi-gene systems analysed later. Let X denote the TF concentration, synthesized with a state-dependent propensity f(X) and degraded at rate αX, resulting in the following reactions:

$$\emptyset \overset{\;f(X)\;\;}{\to }X\;\text{and}\;X\overset{\;\alpha X\;\;}{\to }\emptyset .$$

The corresponding CLE takes the form [16,45]

(2.3) $$dX_{t}=(f(X_{t})-\alpha X_{t})\;dt+\sqrt{f(X_{t})+\alpha X_{t}}dW_{t},$$

where $W_{t}$ is a standard Wiener process. The drift term represents net synthesis and degradation, whereas the diffusion term accounts for stochastic fluctuations inherent in both processes. Setting f(x) equal to one of the Hill functions in equation (2.1) allows us to model activation or repression.

The CLE framework provides a mechanistic description of stochastic gene expression and can be used as a generative model for statistical inference of GRN structure and parameters given a sequence of TF measurements. When discretized using the Euler–Maruyama scheme, the conditional distribution of the state at time $X_{t+\Delta t}$ given the current state $X_{t}$ is Gaussian, with its mean determined by the drift term and variance by the diffusion term [20,39,46,47]. For parameter inference when *network architecture is known*, we can use the discretized version of equation (2.3), which takes the form

(2.4) $$\Delta X=(f(X)-\alpha X)\;\Delta t+\sqrt{\;f(X)+\alpha X\;}\;\sqrt{\Delta t}\epsilon ,$$

where ϵ∼N(0,1) denotes a sample from a standard Gaussian distribution, ΔX=X(t)−X(t−Δt), and we suppressed the explicit dependence on t. This discretization yields a stochastic process, X(t), evolving in discrete time steps of size Δt approximating the continuous-in-time process $X_{t}$. The Euler–Maruyama discretization introduces approximation bias that grows with the sampling interval Δt and with the degree of nonlinearity in the drift and diffusion terms. Since the Hill parameters K and n are fixed to known values, this bias is governed primarily by Δt.

Our goal is also to infer the existence and type of interactions in a GRN. Thus, we will not assume that the architecture of the GRN is known, but will use models that include a range of potential interactions. In the present example, this means that for *inference*, we use the following model, which allows for both activation and repression:

(2.5) $$\Delta X(t)=\left(\frac{\gamma K^{n}}{X^{n}+K^{n}}+\frac{\beta X^{n}}{X^{n}+K^{n}}-\alpha X\right)\Delta t+\sqrt{\frac{\gamma K^{n}}{X^{n}+K^{n}}+\frac{\beta X^{n}}{X^{n}+K^{n}}+\alpha X}\;\sqrt{\Delta t}\epsilon .$$

We assume that elements in the GRN either activate or repress one another or do not interact. We discuss below how a Bayesian approach using equation (2.5) and horseshoe priors for β and γ results in successful inference with estimates $\hat{\beta }=0$ or $\hat{\gamma }=0$, or both.

### 2.3. General inference model

Larger GRNs can be modelled by adding further nodes and interactions. The model we use for inference will always be a generalization of the model used to generate synthetic data that includes a range of potential interactions, e.g. compare equation (2.4) and equation (2.5). In the following, we therefore only describe the model used for inference. We will see that a generalization of equation (2.5) yields explicit transition densities and can be used to define a likelihood function that can be evaluated directly from a sequence of TF measurements. The CLE that approximates the generative model described exactly by the CME also underlies the statistical model used for inference. Hence, the inferred parameters, such as activation or repression rates and degradation constants, retain their meaning in the underlying biochemical reactions.

To extend the inference model to larger GRNs, we represent activation, repression and degradation using matrix-valued functions of the state vector. For simplicity, we suppress the explicit dependence on time in all models defined in this section. If the state of a GRN comprising p genes and the corresponding TFs is given by the vector $X\in R^{p}$, the discretized CLE generalizing equation (2.5) *in the absence of combinatorial regulation* has the form

(2.6) $$\begin{array}{rl} \Delta X & =\underset{\mu (X)}{\underbrace{[\left(\beta ⊙A+\gamma ⊙R\right)1_{p}-\alpha X]}}\Delta t+\underset{\sigma (X)}{\underbrace{\sqrt{\left(\beta ⊙A+\gamma ⊙R\right)1_{p}+\alpha X}}}\;⊙\sqrt{\Delta t}\epsilon , \end{array}$$

where $A:R^{p}\to R^{p\times p}$ and $R:R^{p}\to R^{p\times p}$ are matrix-valued functions. Each matrix element is a Hill function of a single state variable such that $A_{i,j}(X)=f_{a}(X_{j})$ captures the transcription rate of the TF encoded by gene i when regulated by the product of gene j. Similarly, $R_{ij}(X)=f_{r}(X_{j})$ models repression. The matrix $\alpha \in R^{p\times p}$ is diagonal with protein-specific degradation/dilution rates, whereas $\beta ,\gamma \in R^{p\times p}$ contain the maximal transcription rates under activation and repression, respectively. Here and below, the vector $\epsilon \in R^{p}$ consists of p independent samples from the standard Gaussian distribution, and $1_{p}\in R^{p}$ is a vector with all entries equal to 1. In this and subsequent models, μ(X) and σ(X) denote the drift and diffusion terms, respectively.

For example, in a two-gene regulatory network, the parameter matrices take the form

(2.7) $$\begin{array}{rl} \beta & =\left(\begin{array}{cc} \beta_{11} & \beta_{12}\\ \beta_{21} & \beta_{22} \end{array}\right),\;\gamma =\left(\begin{array}{cc} \gamma_{11} & \gamma_{12}\\ \gamma_{21} & \gamma_{22} \end{array}\right),\;\alpha =\left(\begin{array}{cc} \alpha_{1} & 0\\ 0 & \alpha_{2} \end{array}\right),\\ A & =\left(\begin{array}{cc} A_{11}(X_{1}) & A_{12}(X_{2})\\ A_{21}(X_{1}) & A_{22}(X_{2}) \end{array}\right)\;\text{and}\;R=\left(\begin{array}{cc} R_{11}(X_{1}) & R_{12}(X_{2})\\ R_{21}(X_{1}) & R_{22}(X_{2}) \end{array}\right), \end{array}$$

where the elements of the matrices A and R are Hill functions:

$$\begin{array}{r} A_{ij}(X_{j})=\frac{X_{j}^{n}}{K_{j}^{n}+X_{j}^{n}}\;\text{and}\;R_{ij}(X_{j})=\frac{K_{j}^{n}}{K_{j}^{n}+X_{j}^{n}}. \end{array}$$

To capture combinatorial regulation, we extend the model to allow for two regulators per gene via an AND-gate interaction:

(2.8) $$\begin{array}{rl} \Delta X & =\underset{\mu (X)}{\underbrace{[\left(\beta ⊙A+\gamma ⊙R\right)1_{p}+(\eta ⊙H)1_{q}-\alpha X]}}\Delta t+\underset{\sigma (X)}{\underbrace{\sqrt{\left(\beta ⊙A+\gamma ⊙R\right)1_{p}+(\eta ⊙H)1_{q}+\alpha X}}}⊙\sqrt{\Delta t}\epsilon . \end{array}$$

Here, q is the maximum number of regulatory input combinations that a gene can receive, and the matrix $H:R^{p}\to R^{p\times q}$ contains Hill functions representing combinatorial regulation. Each entry $H_{mn}^{i}(X)$ depends on two components of the state vector. For example, in a two-gene regulatory network with possible AND-gate regulation, q=4 corresponds to the four possible two-input combinations so that the matrix H takes the form

$$H=\left(\begin{array}{cccc} H_{aa}^{1}(X_{1},X_{2}) & H_{ar}^{1}(X_{1},X_{2}) & H_{ra}^{1}(X_{1},X_{2}) & H_{rr}^{1}(X_{1},X_{2})\\ H_{aa}^{2}(X_{1},X_{2}) & H_{ar}^{2}(X_{1},X_{2}) & H_{ra}^{2}(X_{1},X_{2}) & H_{rr}^{2}(X_{1},X_{2}) \end{array}\right),$$

where the superscripts denote the gene index and the subscripts, a and r, indicate whether the corresponding regulator is activating or repressing. The functions used in this matrix are defined in equation (2.2).

The parameter matrix $\eta \in R^{p\times q}$ determines the strength of AND gate-mediated regulation. The activation and repression matrices, A and R, and the parameter matrices α,βandγ are defined as in the absence of combinatorial regulation, i.e. as in equation (2.6).

We found that equation (2.8) cannot be used to accurately infer parameters in GRNs with three or more nodes with combinatorial regulation. Convergence and identifiability issues become more pronounced with an increasing number of horseshoe parameters (supplementary material, figure S10). To improve inference in the presence of combinatorial regulation, we thus performed inference in two steps. In the first step, we identify only the presence and absence of regulatory edges. We do so by introducing a matrix of Boolean variables, $\Omega \in \{0,1{\}}^{p\times q}$, whose elements indicate the presence or absence of AND gate-mediated regulation, and a matrix $\theta \in \{0,1{\}}^{p\times p}$ indicating whether a regulatory interaction corresponds to single TF activation or repression. This reduces the number of parameters governed by the horseshoe prior, leading to faster and more stable inference. The resulting model is given by

(2.9) $$\begin{array}{rl} \Delta X & =[\underset{\mu (X)}{\underbrace{\left((\rho \Omega )⊙H\right)1_{q}+\left(\nu ⊙\left(\theta ⊙A+\tilde{\theta }⊙R\right)\right)1_{p}-\alpha X}}]\Delta t+\underset{\sigma (X)}{\underbrace{\sqrt{\left((\rho \Omega )⊙H\right)1_{q}+\left(\nu ⊙\left(\theta ⊙A+\tilde{\theta }⊙R\right)\right)1_{p}+\alpha X}}}⊙\sqrt{\Delta t}\epsilon . \end{array}$$

Here, H,A and R are defined as in equation (2.8). Also, $\tilde{\theta }=1-\theta$ with the difference taken entry-wise. Thus, $\theta_{ij}=1$ indicates the potential presence of an activation of gene i by the product of gene j and the absence of corresponding repression. The matrix $\rho \in R^{p\times p}$ determines the strength of the AND gate-mediated regulation, whereas $\nu \in R^{p\times p}$ represents the strength of direct (single-input) regulatory interactions. The entries in row i of the matrix Ω determine the potential presence of AND gates at gene i: let $\omega_{i,j}\in \{0,1\}$ encode the regulatory role of gene j in the AND-gate pair (j,k) acting on gene i, where $\omega_{ij}=1$ indicates that gene j activates and $\omega_{ij}=0$ indicates that gene j represses the transcription of gene i. Note that j≠k, but either j or k may equal i, allowing for self-regulatory interactions as part of a combinatorial AND gate. Let ${\tilde{\omega }}_{ij}=1-\omega_{ij}$. Then the elements in the ith row of the matrix Ω are the q=2p(p−1) possible pairwise products of all combinations of $\omega_{i,j}$ and ${\tilde{\omega }}_{i,j}$ with differing indices j≠k. Thus, $\omega_{i,j}=\omega_{i,k}=1$ indicates potential activation–activation regulation of gene i by the gene pair (j,k), whereas $\omega_{i,j}={\tilde{\omega }}_{i,k}=1$ indicates potential activation–repression regulation of gene i by the pair (j,k).

In the same example of two genes with possible AND-gate regulation discussed above, the matrix H remains unchanged, while the matrices ρ, Ω andν, and θ take the following forms:

$$\begin{array}{rl} \rho =\left(\begin{array}{cc} \rho_{1} & 0\\ 0 & \rho_{2} \end{array}\right),\;\Omega & =\left(\begin{array}{cccc} \omega_{11}\omega_{12} & \omega_{11}{\tilde{\omega }}_{12} & {\tilde{\omega }}_{11}\omega_{12} & {\tilde{\omega }}_{11}{\tilde{\omega }}_{12}\\ \omega_{21}\omega_{22} & \omega_{21}{\tilde{\omega }}_{22} & {\tilde{\omega }}_{21}\omega_{22} & {\tilde{\omega }}_{21}{\tilde{\omega }}_{22} \end{array}\right),\\ \nu =\left(\begin{array}{cc} \nu_{11} & \nu_{12}\\ \nu_{21} & \nu_{22} \end{array}\right),\;\theta & =\left(\begin{array}{cc} \theta_{11} & \theta_{12}\\ \theta_{21} & \theta_{22} \end{array}\right)\;\text{and}\;\tilde{\theta }=\left(\begin{array}{cc} {\tilde{\theta }}_{11} & {\tilde{\theta }}_{12}\\ {\tilde{\theta }}_{21} & {\tilde{\theta }}_{22} \end{array}\right). \end{array}$$

Here, only one of the entries on each row of Ω is non-zero, since only one of the possible interactions listed in equation (2.2) can be present.

In the second inference step, we infer the reaction rates. To do so, we use the GRN architecture inferred in the first step and the model given by equation (2.8). Assuming a fixed GRN architecture allows us to infer the reaction rates by estimating a substantially reduced number of parameters. We note that the two-step inference procedure is necessitated by identifiability and convergence issues that arise in larger networks with three or more genes. In the two-gene case, the parameter space is sufficiently small that equation (2.9) can be used to simultaneously infer both network architecture (binary indicators $\theta_{ij}\;\text{and}\;\omega_{ij}$) and interaction strengths ($\nu_{ij}$ and $\rho_{i}$) in a single inference step. The two-step procedure is introduced in the context of the three-gene networks considered in §§3.4 and 3.6. In larger networks such as the coherent type-1 feedforward loop (C1-FFL), strong posterior correlations between parameters lead to identifiability issues. In this case, the first step with equation (2.9) is used to determine network topology via a pruning procedure: continuous parameters with a shrinkage factor κ>0.8 and the highest density interval (HDI) concentrated at zero (both with high ESS) are pruned and the corresponding interactions removed, while parameters with moderate κ and HDIs away from zero are retained. The binary indicator variables $\theta_{ij}$ and $\omega_{ij}$ identify the type (activation or repression) of each retained interaction. In the second step, equation (2.8) is applied to the reduced network, retaining only the non-pruned interactions with their inferred regulatory types, and is used to reliably infer the rate parameters of the network.

Thus, we use equation (2.6) for inference under the assumption that each gene has a single regulator. Equation (2.9) is a generalization that we use to allow for possible combinatorial regulation. In the former case, inference requires estimating only continuous parameters (β,γandα), while in the latter, both continuous (ρ,νandα) and discrete (θ and Ω) parameters need to be estimated. These descriptions of the evolution of gene products allow us to define appropriate likelihoods and infer both network structure and kinetic rates from data. We describe this inference framework next.

### 2.4. Bayesian inference framework

We next show how to use Bayesian inference to infer both GRN architecture and reaction rates from data [48]. The Bayes theorem relates model parameter, θ, and the observed measurement, D, via the relation p(θ∣D)∝p(D∣θ)p(θ). In the present case, the likelihood p(D∣θ) is determined by the discretized CLE. Throughout this paper, structural identifiability refers to identifiability at the level of the discretized CLE likelihood, i.e. whether parameters can be uniquely determined from the Gaussian transition densities at the observed time points. Using the Euler–Maruyama approximation, the increments, ΔX, follow a Gaussian distribution with mean determined by the drift term and variance by the diffusion term in the CLE [20]. The likelihood of a full trajectory is then the product of Gaussians evaluated at the observed counts or concentrations across all time steps:

$$\begin{array}{r} p(D|\theta )=\prod_{t=1}^{T-1}N\;(X(t);\;X(t-\Delta t)+\mu (X(t-\Delta t))\Delta t,\;\Sigma^{2}(X(t-\Delta t))\Delta t), \end{array}$$

where N(x;μ,Σ) denotes the probability density function of a Gaussian distribution with mean μ and covariance Σ evaluated at x. Here, the variance matrix is diagonal and equals $\Sigma^{2}(X)=\mathrm{diag}(\sigma^{2}(X))$, where $\sigma^{2}(X)$ is a vector obtained by squaring each component of the diffusion vector σ(X) elementwise. The variance matrix is diagonal because in the underlying biochemical reaction model, the noise terms for different species arise from independent reaction channels. In our model, each reaction channel affects only a single target gene. No reaction channel simultaneously alters multiple species, and stoichiometric coupling between species is therefore absent. As a consequence, cross-species noise correlations do not arise in our formulation, and the diagonal diffusion structure in the CLE is exact for the reaction network considered. However, the framework we describe can be extended to biochemical reaction networks with cross-species coupling. Thus, the increments follow the laws defined by the generative models in equations (2.6)–(2.9).

**Figure 1.**
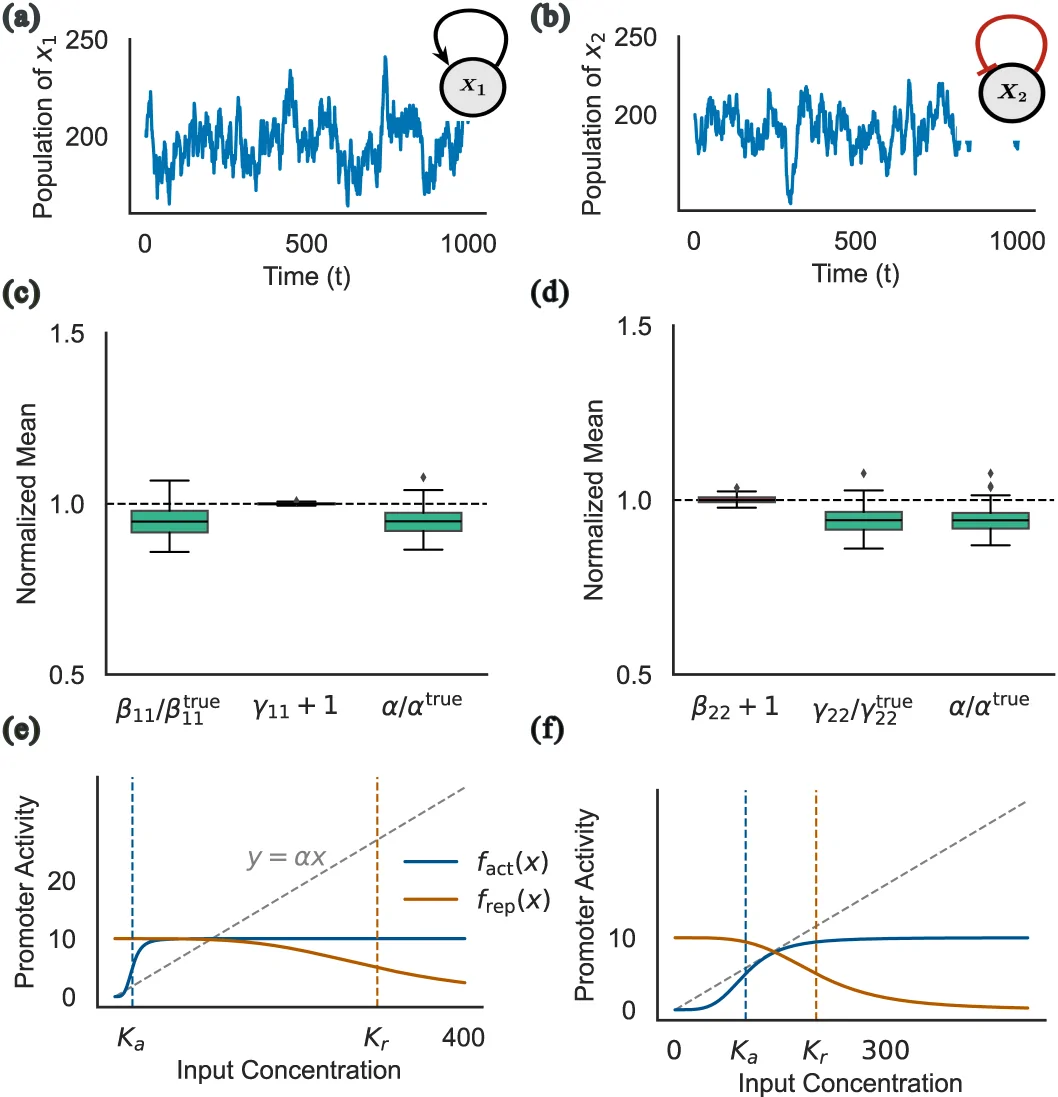
Inference of regulation in a single-gene system. (a,b) TF time series and the corresponding network structures for a single gene: (a) self-activating and (b) self-repressing regulatory network. Each realization was generated using the Gillespie algorithm. Parameters were chosen so that the marginal means and variances of the simulated trajectories were approximately equal for both systems. (c,d) Normalized box plots of the posterior means of the inferred reaction parameters for the (c) self-activating and (d) self-repressing models across 50 realizations. Here, and in the following figures, we show the ratio between the posterior mean and the true value when the true value is non-zero (green), whereas the posterior mean of parameters whose true value is zero is shifted by 1 (red). Hence, estimates close to 1 indicate accurate inference. (e,f) Activation (blue) and repression (red) Hill function in the identifiable (f) and non-identifiable (e) parameter regimes. Detailed results and summary statistics are provided in the supplementary material.

**Parameters estimated.** In all examples, we estimated only the maximal transcription rates and degradation rates, which were consistently identifiable from the data. By contrast, the parameters K (half-maximal concentration) and n (Hill coefficient) were generally more difficult to infer. For instance, as shown in figure 1e, when input concentrations fall within the saturated region of the response curve, the system becomes insensitive to changes in K, rendering this parameter difficult to identify. By contrast, figure 1f illustrates that when the input spans the dynamic range around K, its influence on the output becomes observable, enabling more reliable inference. Even in such regimes, inference of K remained sensitive to prior assumptions; good estimates required using informative priors. The Hill coefficient, n, was typically even more weakly identifiable. It is known that K and n are often structurally or practically unidentifiable, as they do not strongly determine the system's dynamics [49,50]. We thus fixed both parameters to their known values during inference. With K and n fixed, the maximal transcription rates (β, γ, ν and ρ) become consistently estimable, provided that TF concentrations span the dynamic range of the Hill function (figure 1f); when K and n are free, only the ratio $\beta /K^{n}$ is identifiable at low TF concentrations. All synthetic data used in this work were generated in this identifiable regime (see supplementary material, §7, for a detailed identifiability analysis).

**Priors.** We used uninformative priors in all examples. These priors could be changed to include available information about the structure of the GRN or reaction rates. We distinguish two cases corresponding to the two model formulations:

—*Single-regulator model (no combinatorial regulation) (equation (2.6)).* Reaction rates are determined by the activation strengths, β, repression strengths, γ, and degradation rates, α. To promote sparsity in interactions, we used horseshoe priors for β and γ [18]. These priors reflect the need for strong evidence for an interaction, and the assumption that a TF cannot both activate and repress the same target, i.e. that for each gene pair, (i,j), at most one of $\beta_{ij}=0$ and $\gamma_{ij}=0$ is non-zero. These shrinkage priors strongly pull most interaction strengths towards zero while allowing a subset to remain large, consistent with the expectation that only a few of all possible interactions are present in GRNs.

—*Combinatorial model (equation (2.9)).* In addition to single-input activation and repression regulatory strengths in the parameter matrix ν and degradation rates α, this model includes the gate-strength matrix, ρ, as well as a matrix of binary indicator variables, θ, corresponding to activation/repression and Ω corresponding to AND-gate regulation. We used regularized horseshoe priors for parameters ρ and ν to reflect the assumption of sparsity in both gated and non-gated interactions. The binary indicators are assigned independent, uninformative Bernoulli(0.5) priors, representing the prior belief that each potential interaction or gate is equally likely to exist or not. The binary indicator variables appear in complementary pairs, ${\tilde{\theta }}_{ij}=1-\theta_{ij}$ and ${\tilde{\omega }}_{ij}=1-\omega_{ij}$, ensuring that the continuous parameters $\nu_{ij}$ and $\rho_{i}$ always appear in the likelihood and remain identifiable. When $\nu_{ij}$ or $\rho_{i}$ is inferred to be near zero under the horseshoe prior, the corresponding interaction or AND gate is excluded from the network. However, strong posterior correlations between AND-gate and single-input parameters at the same gene reflect a structural identifiability issue. This motivated the two-step inference procedure, in which network architecture is identified before rate parameters are estimated, substantially reducing parameter correlations and improving sampler convergence.

For the degradation rates, α, which are always positive, we used uninformative half-normal priors. This enforces non-negativity while constraining the inferred values to the expected range of degradation rates.

To improve sampling efficiency and avoid issues related to thick Cauchy tails when using horseshoe priors, we adopted the recommendations by Piironen & Vehtari [18] and replaced the half-Cauchy priors with half-Student-t distributions. Specifically, we set

$$\begin{array}{rl} \psi |{\tilde{\lambda }}_{i},\tau ,c & ∼N\left(0,{\tilde{\lambda }}_{i}^{2}\tau^{2}\right)\\ {\tilde{\lambda }}_{i} & =\frac{c\lambda_{i}}{\sqrt{c^{2}+\lambda_{i}^{2}\tau^{2}}}\\ \lambda_{i} & ∼\text{half-Student-}t(1,1)\\ c^{2} & ∼\text{inv-gamma}\left(\frac{\nu }{2},\frac{\nu s^{2}}{2}\right)\\ \tau & ∼\text{half-Student-}t(1,0.1). \end{array}$$

Here, ψ represents parameters for which we used the horseshoe prior. Following standard practice, we chose v=4 and s=2 for the inverse-gamma prior on $c^{2}$. This formulation provides robust shrinkage, ensures well-behaved posteriors and facilitates efficient posterior sampling for high-dimensional or weakly identified models [18,21].

**Approximating the posterior distributions.** Samples from the posterior distributions were generated using PyMC [51,52]. For models with only continuous parameters, we used Hamiltonian Monte Carlo (HMC) [53] with the No-U-Turn Sampler (NUTS) [54], accelerated via the BlackJAX backend for efficient sampling on CPUs and GPUs. When discrete parameters were present (e.g. indicator matrices), we reverted to PyMC's standard NUTS implementation, which marginalizes over discrete variables when possible. Convergence was assessed using standard diagnostics, including the $\hat{R}$ statistic, effective sample size and visual inspection of trace plots. In models with discrete indicator variables, PyMC samples binary variables using a Gibbs binary Metropolis–Hastings step and continuous parameters using HMC/NUTS. Convergence was well behaved across all examples, with $\hat{R}\leq 1.05$ and effective sample sizes within acceptable ranges. The only mixing challenges arose in the C1-FFL example, where a structural non-identifiability between the AND-gate and single-input strengths produced slow mixing along that direction, while the binary indicators and pruned parameters mixed well. These were mitigated by the two-step inference procedure, which reduced the effective dimensionality of the parameter space in the second step.

### 2.5. Synthetic data

We inferred parameters using TF measurements from a single trajectory at a stationary equilibrium. The likelihood is constructed as a product of Gaussian transition densities over consecutive time steps within the trajectory. This is distinct from cross-sectional single-cell data, where only snapshots of the marginal distribution are available. Such data would be insufficient, as our approach relies on the temporal autocorrelation structure in the observations to distinguish regulatory modes.

We generated synthetic gene-expression trajectories using the SSA [17,55]. In all simulations, an initial burn-in period was discarded, chosen by monitoring the running mean and variance of TF counts until stabilization. We chose the sampling interval Δt to be sufficiently small so that the assumed discretization provides a good approximation of the underlying dynamics, and sufficiently large so that increments in TF numbers are approximately Gaussian and the correlation between consecutive observations is not excessive. For each parameter setting, we generated 50 independent realizations, each consisting of 3000 measurements. Replicate datasets were generated to ensure robust inference and allowed us to account for variability across stochastic sample paths [4]. All SSA simulations were implemented using the Python package GillesPy2 [56].

## 3. Results

GRNs govern cellular behaviour through transcriptional regulation, with TFs activating or repressing target genes. Inferring GRN structure and dynamics from gene expression data is challenging owing to the complexity of regulatory interactions and the stochasticity of gene expression. We show that the proposed Bayesian inference framework allows us to simultaneously recover network topology, regulatory modes (activation/repression) and kinetic parameters from time-series expression data.

We demonstrate the performance of the proposed inference method using synthetic data from a range of canonical gene regulatory motifs. Throughout, we consider GRNs at stochastic equilibrium, with molecular fluctuations having reached their stationary probability distribution. ODE and logic-based models cannot capture stationary fluctuations in molecule numbers [57]. Structural inference of GRNs using such models, therefore, requires the observation of transients. By contrast, we show that a generative model that accounts for intrinsic stochasticity allows for the inference of kinetic parameters and regulatory structure from measurements of GRNs at stochastic equilibrium.

We illustrate the proposed method using a sequence of examples of increasing complexity. We begin with single-gene autoregulation, then consider the classic two-gene toggle switch [3], followed by an extension to a toggle with combinatorial regulation. We next consider the three-gene repressilator [58], a canonical oscillatory circuit, and finally turn to a C1-FFL [30,59]. This allows us to assess both the accuracy and the scalability of the framework.

Throughout, we work in rescaled (non-dimensional) time, so all parameters are expressed without physical units. Under this convention, the transcription rate parameters correspond to the maximal expected number of molecules transcribed per unit rescaled time. In all examples, we performed inference on 50 independent realizations to quantify robustness. We also report posterior means (denoted by $\hat{\psi }$ for a parameter ψ) and HDIs for a single, representative realization. Here, HDI denotes the highest density interval: the narrowest interval containing the stated posterior mass, reported at the 94% level; bounds are labelled hdi_3%/hdi_97%. We show representative results here, while the full inference results along with a complete description of the models used for inference are provided in the supplementary material.

### 3.1. Single-gene motifs

We first consider a single-gene autoregulatory circuit. In this motif, the product of a gene regulates its own production, as shown in figure 1a,b. Here, and in all subsequent examples, black edges represent activation and red edges represent repression. We consider both self-activation and self-repression, with transcription rates defined by Hill functions given in equation (2.1). Details about the parameters used to generate the synthetic data for this and subsequent examples are provided in the supplementary material.

We first asked whether we could use the proposed inference framework to distinguish between activation and repression from trajectories in stochastic equilibrium. To do so, we generated synthetic TF trajectories for both cases using parameters that produced marginal distributions with matched means and variances. Example trajectories are shown in figure 1a,b. The associated marginal distributions of TF counts are nearly indistinguishable (supplementary material, figure S1). Many existing inference approaches, such as moment-matching [19] and fitting to the steady-state distribution [60], use only marginal statistics and would therefore fail to distinguish activation and repression from such data.

Despite the similarity in marginal distributions, we successfully inferred the correct regulatory mode in all 50 independent realizations. In the activation case, the repression parameter $\gamma_{11}$ was estimated to be near zero (posterior mean ${\hat{\gamma }}_{11}=0.001,\;\text{HDI:}\;[-0.95,\;1.05])$, whereas the activation parameter $\beta_{11}$ was inferred to be close to its true value of 10 $\left({\hat{\beta }}_{11}=9.07,\;\text{HDI:}\;[8.31,\;9.87]\right)$. Conversely, in the repression case, the activation parameter $\beta_{22}$ was inferred to be near zero $\left({\hat{\beta }}_{22}=-0.003,\;\text{HDI:}\;[-1.02,\;0.94]\right)$, whereas the repression parameter $\gamma_{22}$ was estimated to be close to 10 $\left({\hat{\gamma }}_{22}=9.56,\;\text{HDI:}\;[8.74,\;10.40]\right)$ (see supplementary material, table S4, for exact values). Figure 1c,d shows the normalized box plots of the inferred parameters for self-activation and self-repression across 50 independent SSA realizations.

We distinguish two fundamentally different sources of information available at the stochastic equilibrium. Methods based on stationary marginals, such as moment-matching or fitting to the steady-state distribution, use only the time-averaged statistics of TF counts (means, variances and higher moments), discarding all temporal structure. As demonstrated in the single-gene example (figure 1; supplementary material, figure S1), self-activation and self-repression can produce identical marginal distributions, so marginal-based methods cannot distinguish these regulatory modes. By contrast, our approach exploits the full stationary path law: the CLE likelihood is constructed from transition densities between consecutive time points, capturing the temporal autocorrelation structure and increment statistics of the fluctuations. These temporal features differ between activation and repression even when marginal statistics are matched, enabling the discrimination of regulatory modes that are invisible to marginal-based approaches [61].

However, we found that the system is not identifiable in the *saturated regimes* of the Hill function (figure 1e). At sufficiently high or very low concentrations of the TF, the Hill function saturates, and fluctuations in counts of a regulating TF have a negligible effect on the production rate. In this regime, the likelihood function is approximately constant, making it difficult to distinguish between activation and repression and infer the transcription rates. This limitation is well recognized: in saturated regimes, different Hill functions can produce indistinguishable observable statistics [31]. A likelihood-based inference framework helps to determine when such identifiability issues arise [62] and determine the parameter regimes in which the likelihood is sufficiently sensitive to changes in TF count to make inference possible (figure 1f). In all the examples discussed in this text, we therefore work with synthetic data generated in this regime.

### 3.2. Two-gene motifs

**Figure 2.**
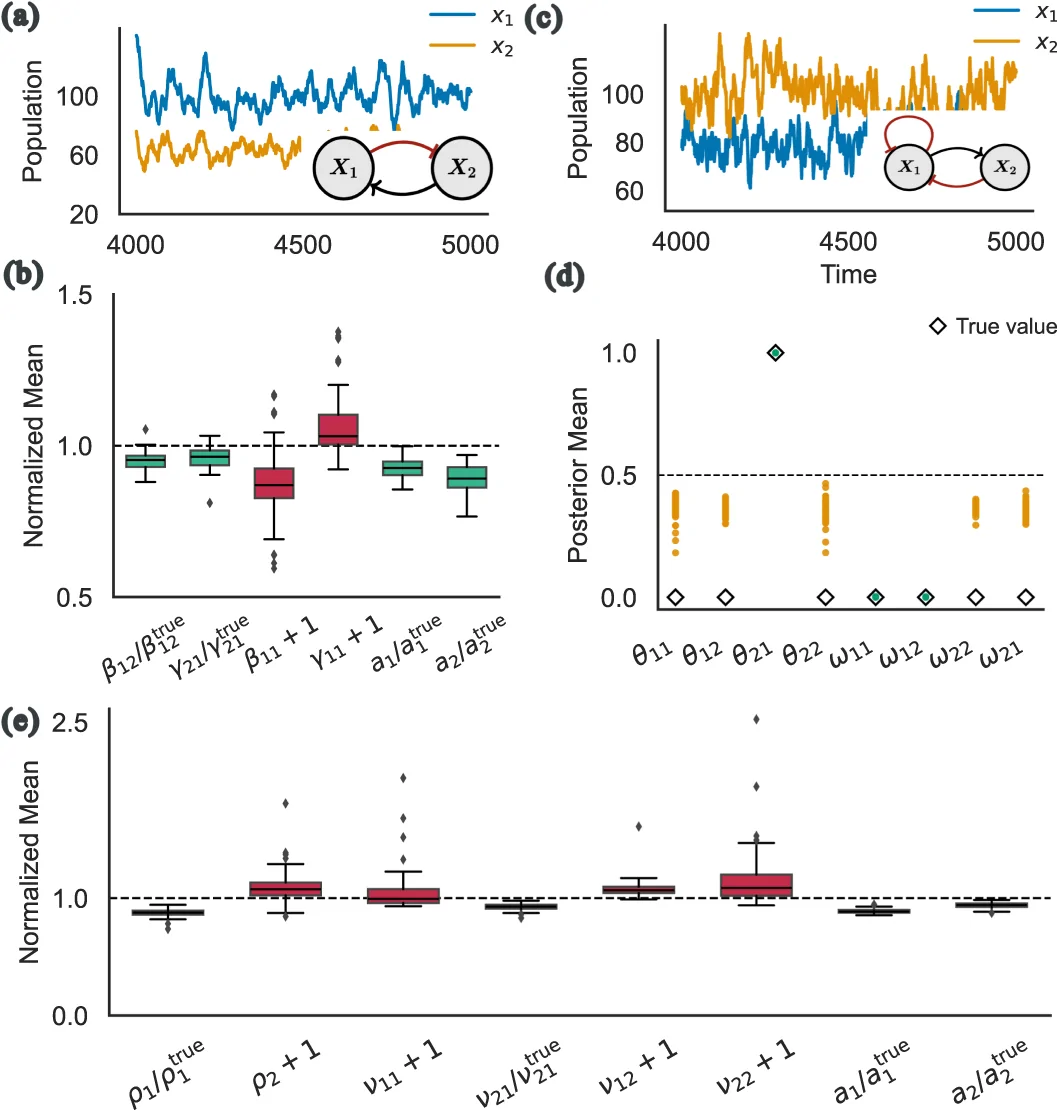
Inference in two-gene regulatory networks with and without combinatorial regulation. (a) Expression levels and corresponding network structures for a two-gene system without combinatorial regulation. (b) Normalized box plots of the posterior means of the inferred parameters obtained from 50 realizations of the trajectories. (c) Expression levels for a two-gene regulatory system with combinatorial AND-gate regulation. (d) Posterior means of the inferred binary regulatory indicators (θ, ω) obtained from 50 time-series realizations. We used a threshold of 0.5 to determine the presence and type of regulatory interactions. (e) Normalized posterior means of the inferred reaction rates. Here and in subsequent figures, we present results for a representative subset of key parameters; detailed results are provided in the supplementary material.

We next consider a two-gene toggle-switch-type network, in which the product of gene 2 activates gene 1 while gene 2 is repressed by the product of gene 1 as shown in figure 2a. Such canonical motifs have been studied in both natural regulatory circuits and synthetic gene networks [63,64]. We generated synthetic data from the GRN shown in figure 2a and modelled the system using equation (2.6), where the two-gene case is provided as an illustrative example (see equation (2.7) for the corresponding matrices). For inference, we used a fully connected GRN model that encompasses all potential interactions (see supplementary material, equation (2), for the definition of the likelihoods).

We again accurately recovered the underlying regulatory network in 50 independent realizations. The activation parameter $\beta_{12}$ (true value = 10) was inferred to be close to its true value $\left({\hat{\beta }}_{12}=9.18,\;\text{HDI:}\;[8.14,\;10.16]\right)$, correctly identifying the product of gene 2 as an activator of gene 1. Likewise, the repression parameter $\gamma_{21}$ (true value = 20) was estimated to be close to 20 $\left({\hat{\gamma }}_{21}=20.212,\;\text{HDI:}\;[18.08,\;22.33]\right)$, accurately capturing the repressive interaction. The degradation parameters $\alpha_{1}$ and $\alpha_{2}$ were also accurately estimated (${\hat{\alpha }}_{1}=0.028,\;\text{HDI:}\;[0.022,\;0.031]$ and ${\hat{\alpha }}_{2}=0.018,\;\text{HDI:}\;[0.013,\;0.022]$, respectively), corresponding to relative errors below 5%. All other regulatory parameters $\beta_{11}$ and $\gamma_{11}$ corresponding to non-existent interactions had posterior means below 0.2, with 95% HDIs restricted to values near zero. Hence, we did not identify any spurious interactions.

These inference results are summarized in figure 2b, which demonstrates that we can accurately infer both the structure of the GRN and the parameters governing TF dynamics at a stochastic equilibrium.

### 3.3. Two-gene motifs with an AND gate

We next considered the case of a two-gene motif with both cross-regulation and autoregulation shown in figure 2c. Such network motifs, combining cross-feedback with autoregulation, are common in both natural and synthetic gene networks [65] and can generate rich dynamical behaviours including oscillations and bistability [66,67]. We again focus on the non-oscillatory regime.

This circuit poses a stringent test for network inference because autoregulatory interactions can be obscured by cross-regulatory signals and because self-regulation produces temporal correlations that can confound parameter estimation [68]. Successful recovery of both auto- and cross-regulatory interactions in this motif thus demonstrates the ability to disentangle multiple regulatory modes acting on the same gene.

We used synthetic trajectories from a system in which gene 1 was regulated by the product of gene 2 and by its own product, as shown in figure 2c. Regulation was modelled using AND-gate logic according to equation (2.9). For inference, we used equation (2.9) instead of equation (2.8) because it is computationally more efficient, requiring the inference of fewer parameters.

For inference, we again used a fully connected GRN model that included all potential interactions, including autoregulation (supplementary material, equation (4)). Binary-valued variables in the matrices Ω and Θ indicate whether potential regulatory interactions were activating or repressive, while continuous-valued parameters (ρ,ν) quantified the interaction strength (§2.3). When a continuous parameter was inferred to be close to zero (i.e. when the corresponding HDI is concentrated around 0), the corresponding edge was assumed to be absent. Thus, edge existence and interaction strengths were inferred simultaneously within a single inference step. By contrast to the larger networks considered in §§3.4 and 3.6, the two-gene network is sufficiently small that edge existence and interaction strengths can be inferred simultaneously using equation (2.9) in a single inference step, without the need for the two-step procedure.

We were able to infer the correct two-gene AND-gate motif structure from all 50 independent synthetic trajectories. The scatter plots in figure 2d,e show the posterior means for the binary variables and continuous variables, respectively. In this example, the presence of regulatory interactions is determined by the product of the corresponding binary and continuous variables, specifically through the terms $\theta_{ij}\nu_{ij}$ and $\rho_{i}\omega_{ij}\omega_{ik}$, which becomes negligible when either component is inferred to be close to zero. In figure 2d, orange dots represent binary variables associated with parameters whose true value is zero.

Since gene 1 represses its own production and is repressed by $X_{2}$, its reaction is described by a repression–repression term, $\rho_{1}({\tilde{\omega }}_{11})({\tilde{\omega }}_{12})H_{rr}^{1}$. Green dots in figure 2d correspond to ${\hat{\omega }}_{11},{\hat{\omega }}_{12}$ being close to zero, indicating the presence of combinatorial AND-gate repression at gene 1. The parameter $\rho_{1}$ is inferred to be close to its true value of 20 $\left({\hat{\rho }}_{1}=18.27,\;\text{HDI:}\;[16.81,\;19.73]\right)$, while the parameters $\theta_{11}$ and $\theta_{12}$ are estimated to be close to zero (${\hat{\theta }}_{11}$=0 and ${\hat{\theta }}_{12}=0$). The corresponding parameters, $\nu_{11}$ and $\nu_{12}$, are also inferred to be close to zero $\left({\hat{\nu }}_{11}=-0.04,\;\text{HDI:}\;[-0.61,\;0.48\right]$ and ${\hat{\nu }}_{12}=0.09,\;\text{HDI:}\;[-0.76,\;1.02])$ (figure 2e), indicating the absence of the corresponding edges in the GRN.

Gene 2 transcription is activated by the product of gene 1, which is captured by the term $\nu_{21}\theta_{21}A_{21}$. As shown in figure 2d,e, green dots correspond to the estimate ${\hat{\theta }}_{21}\approx 1$. The related parameter $\nu_{21}$ indicates a strong activating effect of the product of gene 1 on gene 2 $\left({\hat{\nu }}_{21}=9.42,\;\text{HDI:}\;[8.82,\;10.03]\right)$. On the other hand, the parameters $\nu_{22}$ $\left({\hat{\nu }}_{22}=0.03,\;\text{HDI:}\;[-0.65,\;0.78]\right)$ and $\rho_{2}$ $\left({\hat{\rho }}_{2}=0.02,\;\text{HDI:}\;[-0.84,\;0.94]\right)$ were inferred to be close to zero, indicating the absence of self-regulation and combinatorial regulation at gene 2.

Thus, the inferred GRN structure and parameters were consistent with the motif structure we used to generate the data, and we were able to infer both the existence of combinatorial regulation and the strengths of the underlying interactions in the presence of multiple regulatory inputs.

### 3.4. Repressilator

**Figure 3.**
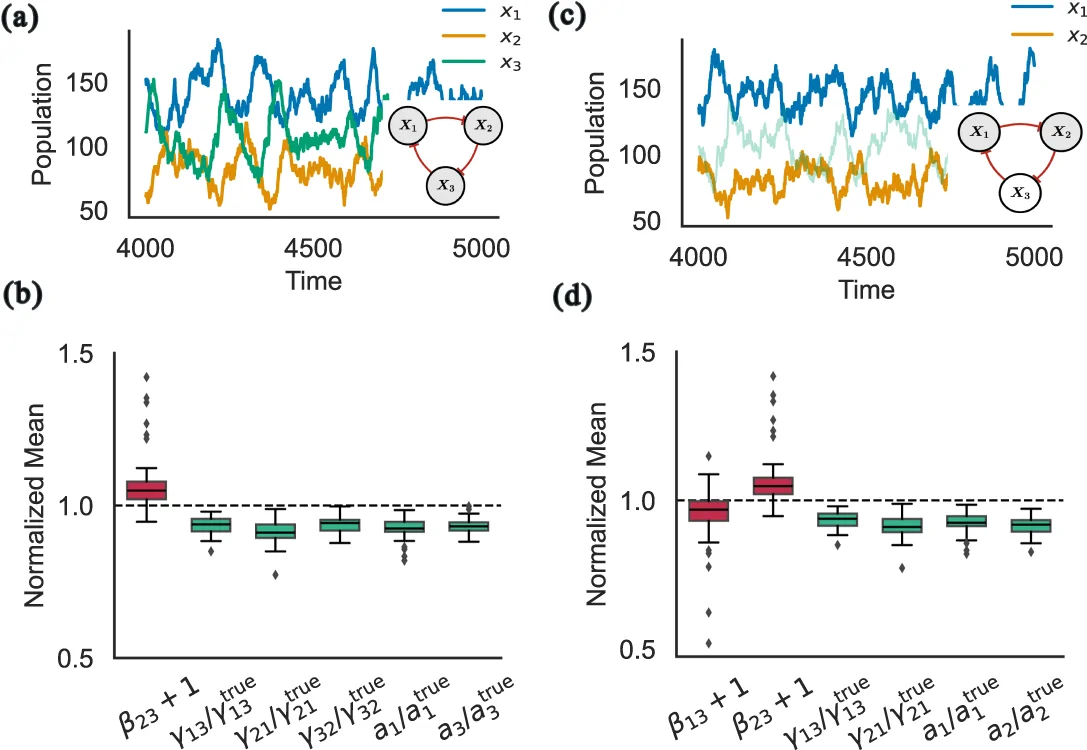
Inference in the non-oscillatory repressilator. (a) Trajectories of TF levels in a fully observed repressilator system ($X_{1}⊣X_{2}⊣X_{3}⊣X_{1}$). (b) Normalized box plots of the posterior means of inferred regulatory parameters obtained from 50 realizations of the trajectories. (c) The same repressilator system when only partially observed with the product of gene 3 (light green line) was not used for inference. (d) The normalized box plots of the inferred parameters show that we recovered the correct regulatory topology even when the GRN is only partially observed.

We next considered the repressilator [69], a synthetic circuit that allows us to test the performance of the proposed inference methods with cyclic regulatory architectures. The repressilator consists of three transcriptional repressors arranged in a cyclic inhibitory loop [58], as shown in figure 3a. While this network can generate sustained oscillations, we focused on the non-oscillatory regime, where trajectories converge to a stochastic equilibrium.

We were again able to both identify the correct regulatory structure and accurately estimate the maximal transcription rates, as well as degradation rates from synthetic data. The box plots in figure 3b show that the recovered parameters closely match the ground truth. Across 50 independent realizations, the repression parameters $\gamma_{13}$, $\gamma_{21}$ and $\gamma_{32}$ were inferred with posterior mean estimates close to their true values of 10 (${\hat{\gamma }}_{13}=9.485,\;\text{HDI:}\;[8.43,\;10.36]$), 20 (${\hat{\gamma }}_{21}=18.287,\;\text{HDI:}\;[16.53,\;19.86]$) and 15 (${\hat{\gamma }}_{32}=14.197,\;\text{HDI:}\;[13.20,\;15.15]$), respectively. All other interaction parameters (${\hat{\beta }}_{ij}$ and ${\hat{\gamma }}_{ij}$) were estimated to be close to zero, correctly indicating the absence of other interactions. The degradation rates, ${\hat{\alpha }}_{1}$, ${\hat{\alpha }}_{2}$ and ${\hat{\alpha }}_{3}$, were also recovered accurately.

### 3.5. Partial observability

In practice, we can often observe only a subset of a GRN. This poses significant challenges for network inference and parameter identifiability [70]. To evaluate our method's performance under partial observability, we returned to the three-gene repressilator motif but assumed that only the products of genes 1 and 2 are measurable (figure 3c). This allowed us to test what interactions can be inferred when the network is partially observed.

Despite the absence of direct measurements of the product of gene 3, we recovered all parameters governing the dynamics of the *observed* nodes. We were able to infer that the product of gene 1 represses gene 2, with $\gamma_{21}$ estimated close to its true value of 20 $\left({\hat{\gamma }}_{21}=18.28,\;\text{HDI:}\;[16.46,\;19.84]\right)$, and that the product of gene 3 represses gene 1, with $\gamma_{13}$ estimated near its true value of 10 $\left({\hat{\gamma }}_{13}=9.49,\;\text{HDI:}\;[8.43,\;10.34]\right)$. The degradation rates were also inferred accurately, with ${\hat{\alpha }}_{1}=0.03,\;\text{HDI:}\;[0.027,\;0.032]$ and ${\hat{\alpha }}_{2}=0.05,\;\text{HDI:}\;[0.042,\;0.050]$. All other parameters governing the evolution of $X_{1}$ and $X_{2}$ were also estimated well. The horseshoe prior effectively suppressed spurious interactions, with the estimated activation parameters $\beta_{13}$ and $\beta_{23}$ taking posterior means near zero $\left({\hat{\beta }}_{13}=0.018,\;\text{HDI:}\;[-0.29,\;0.38\right]$ and ${\hat{\beta }}_{23}=0.088,\;\text{HDI:}\;[-0.24,\;0.62])$, respectively, indicating the absence of the corresponding regulatory effects.

As expected, parameters determining the dynamics of products of the unobserved gene 3, including ${\hat{\gamma }}_{32}$ and ${\hat{\alpha }}_{3}$, had large posterior uncertainty. However, the parameter $\gamma_{13}$ captures the regulatory impact of the unobserved gene 3 on the observed system dynamics and was inferred correctly. Thus, our method correctly inferred the presence and strength of this hidden regulatory link without generating false positive interactions, demonstrating robust performance under partial observability.

### 3.6. Coherent type-1 feedforward loop

The C1-FFL is one of the most frequently occurring regulatory motifs in transcriptional networks [30,59]. It consists of a regulator that activates a downstream target both directly and indirectly through an intermediate regulator, with all three interactions being activating. C1-FFLs generate *sign-sensitive delays*, in which activation of the target requires sustained input. This property enables the motif to filter out transient fluctuations and contributes to the robustness of gene expression [59].

**Figure 4.**
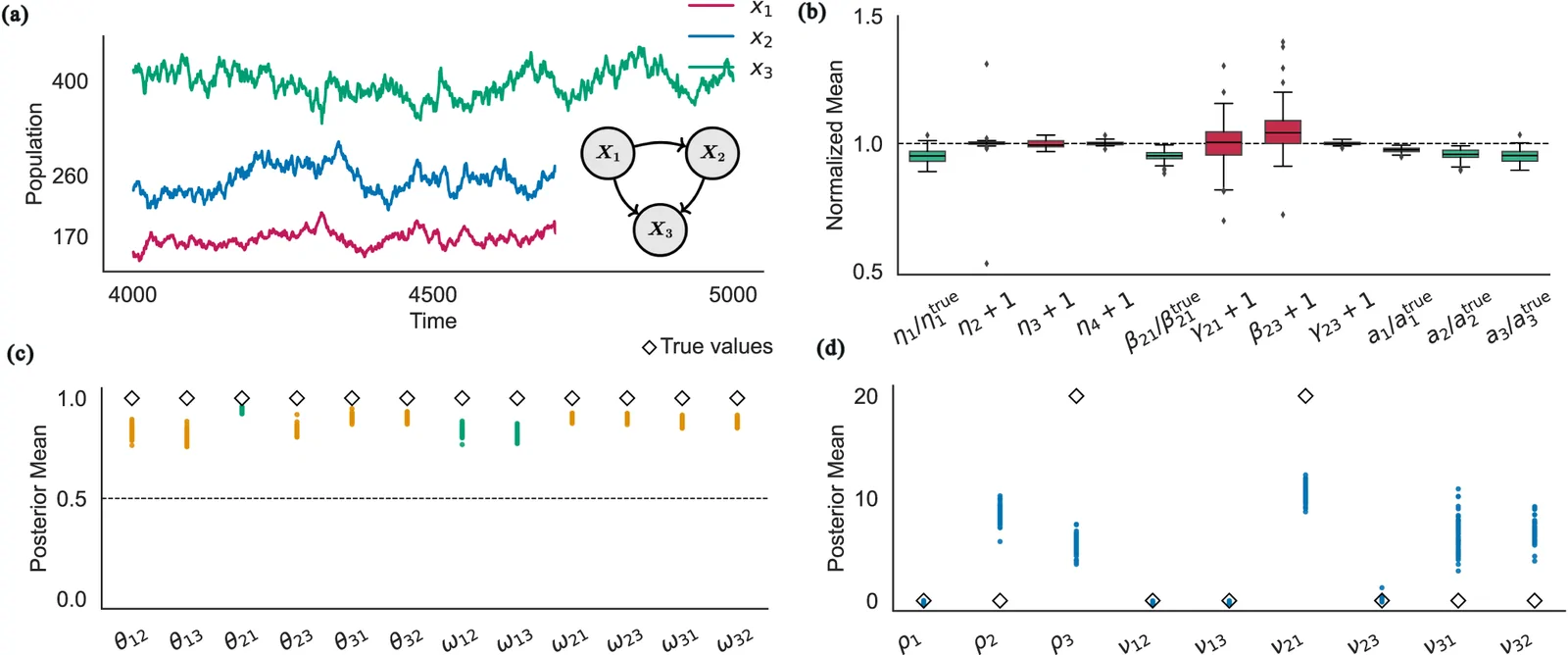
Results of the two-step inference procedure applied to the C1-FFL motif. (a) Gene expression trajectories generated of a network motif where gene $X_{1}$ is produced at a constant rate of 5 units, activates $X_{2}$, and $X_{3}$ is jointly activated by both $X_{1}$ and $X_{2}$ through an AND gate. (b) Normalized box plots from the second inference step, showing the ratios of posterior means to true parameter values for β (activation strength), α (degradation rate) and η (combinatorial regulation strength). (c) Scatter plot of inferred binary regulatory indicators (θ andω) from the first inference step, where a threshold of 0.5 was used to determine the presence and type of regulatory interactions. The threshold of 0.5 for the binary indicator variables $\theta_{ij}$ and $\omega_{ij}$ follows naturally from the Bernoulli (0.5) prior, which reflects equal prior probability for activation or repression. A posterior mean above 0.5 indicates activation, while a posterior mean below 0.5 indicates repression, corresponding to a MAP decision rule under this symmetric prior. (d) Scatter plots of the inferred continuous parameters (ν andρ) from the first inference step, representing the strength of individual and combinatorial regulatory interactions.

To model this GRN, we assumed that gene 1 is transcribed at a constant rate, and the product of gene 1 activates the expression of gene 2, while both the products of gene 1 and 2 jointly activate the expression of gene 3 as shown in figure 4a. In the model used for inference, we did not allow for self-loops as they significantly increased the number of possible combinations of interactions at each gene. Although they could be incorporated, doing so would considerably increase computational demands. We performed inference in two steps because jointly inferring GRN structure and transcription rates with equation (2.9) is unreliable. The difficulty arises because equation (2.9) contains a structural non-identifiability between the AND-gate strength $\rho_{i}$ and the single-input strengths $\nu_{ij}$ at genes receiving both forms of regulation: their sum is constrained by the data, but their individual values are not. This redundant direction yields a sharply curved posterior geometry along which the HMC/NUTS sampler diverges. The divergences are confined to this direction: divergent draws scatter across the wide ridge spanned by $\rho_{i}$ and $\nu_{ij}$, while the binary indicators and pruned continuous parameters on which the architecture decisions rest are unaffected (posterior summaries at divergent and non-divergent draws agree to within sampling error, with bulk-ESS in the thousands to tens of thousands). The non-identifiable parameters mix slowly (bulk-ESS in the low hundreds), with wide, overlapping HDIs; their means sum to recover the true regulatory total at each gene (supplementary material, table S13). The two-step procedure resolves this by estimating rates on the reduced model (equation (2.8)), which retains only the supported regulatory terms and so no longer admits the $\rho_{i}/\nu_{ij}$ trade-off. Sampling performance improved substantially even over the course of this project as the HMC/NUTS implementations in PyMC were refined (see Conclusion).

The first inference step was focused on inferring GRN architecture. Here, we used equation (2.9) to define the likelihood of the model parameters. This formulation of the generative model incorporates binary indicator variables to identify active regulatory interactions. When the associated continuous parameters had HDIs concentrated around zero, the corresponding interactions were assumed to be absent. Pruning these potential interactions yielded a reduced model that we used in the second inference step focused on inferring interaction strengths and degradation rates. Specifically, continuous parameters with shrinkage factors κ>0.8 and posterior mass concentrated near zero are pruned, as they indicate strong regularization under the horseshoe prior, while the binary indicators $\theta_{ij}$ and $\omega_{ij}$ from the first step determined the type (activation or repression) of each retained interaction. The second step then used equation (2.8) with this substantially reduced parameter space. This two-stage approach reduced the effective dimensionality of the parameter space and mitigated the influence of spurious edges on rate estimates.

In the first step across 50 realizations, the model consistently inferred ${\hat{\theta }}_{12}=1$, ${\hat{\theta }}_{23}=1$, ${\hat{\theta }}_{21}=1$, ${\hat{\theta }}_{13}=1$, ${\hat{\theta }}_{31}=1$ and ${\hat{\theta }}_{32}=1$, indicating that all six potential interactions were present (figure 4c). The AND-gate binary indicator variables $\omega_{ij}$ were also inferred to be active: ${\hat{\omega }}_{12}=1$, ${\hat{\omega }}_{13}$=1, ${\hat{\omega }}_{21}=1$, ${\hat{\omega }}_{23}=1$, ${\hat{\omega }}_{31}=1$ and ${\hat{\omega }}_{32}=1$.

As shown in figure 4d, the AND-gate strengths were estimated as ${\hat{\rho }}_{1}=0,\;\text{HDI:}\;[-0.71,\;0.36]$ and ${\hat{\rho }}_{2}=8.88,\;\text{HDI:}\;[-0.61,\;20.96]$, suggesting no AND-gate regulation at gene 1 but a potential combinatorial effect at gene 2. The single-input parameters acting on gene 2 were inferred as ${\hat{\nu }}_{21}=11.12,\;\text{HDI:}\;[-0.51,\;20.97]$ and ${\hat{\nu }}_{23}=-0.08,\;\text{HDI:}\;[-1.36,\;0.74]$. We note that it is not $\nu_{21}$ being non-zero alone that rules out the AND gate at gene 2, but rather the combination of $\nu_{21}$ being non-zero and $\nu_{23}\approx 0$. Although there is a structural identifiability issue between $\rho_{2}$ and $\nu_{21}$, where the regulatory strength is split between the two terms, $\nu_{23}\approx 0$ conclusively rules out any regulatory role for gene 3 to gene 2 in either the single input or AND-gate term. Since an AND gate requires two non-zero inputs, the AND gate at gene 2 is ruled out. This contrasts with gene 3, where all three parameters $\rho_{3}$, $\nu_{31}$ and $\nu_{32}$ are non-zero with wide overlapping HDIs, reflecting the splitting of regulatory strength between the AND gate and single-input terms, which is resolved by the two-step inference procedure.

For gene 3, $\rho_{3}$ was inferred to be non-zero (${\hat{\rho }}_{3}=5.27,\;\text{HDI:}\;[-0.66,18.79]$), and the corresponding single-input parameters, estimated as ${\hat{\nu }}_{31}=5.96,\;\text{HDI:}\;[-0.54,\;18.73]$ and ${\hat{\nu }}_{32}=6.72,\;\text{HDI:}\;[-0.51,\;18.95]$, had wide HDIs that included substantial non-zero values, indicating an AND-gate interaction involving TFs $X_{1}$ and $X_{2}$. In the second step, only the AND-gate regulation of $X_{3}$ was retained, with the single-input parameters $\nu_{31}$ and $\nu_{32}$ pruned to zero. Although the single-input terms had positive estimated values, they were pruned because the strong interaction term $\rho_{3}$ alone was sufficient to explain the data. This resulted in a simpler and more interpretable model, in which gene 3 is activated exclusively by the simultaneous presence of both TFs $X_{1}$ and $X_{2}$, with no independent activation by either factor alone.

The final inference results demonstrate that the structure of the GRN and the interaction strengths were accurately recovered (figure 4b). Thus, a two-step approach consisting of first identifying the GRN structure and then estimating the corresponding rate parameters can improve inference in complex networks, enabling both accurate identification of network architecture and parameter estimation.

## 4. Discussion

We have shown that the CLE provides a tractable and biologically grounded framework for inferring network structure and kinetic parameters in GRNs operating in the mesoscopic regime, where intrinsic stochasticity is significant but molecule counts are sufficiently large to justify a continuous approximation. The CLE captures both deterministic reaction kinetics and stochastic fluctuations within a unified stochastic differential equation framework, making it well suited for modelling gene expression, where noise plays important functional roles [36,71]. A comparison with a linearized VAR(1) baseline (supplementary material, S8) confirms that the full nonlinear CLE outperforms inference using a linear model when regulatory interactions are strong or when activation and repression must be distinguished. While exact likelihood-based inference using the CME is theoretically optimal, it becomes computationally intractable for multi-gene networks [72]. The CLE offers a practical alternative that retains essential stochastic information while remaining amenable to efficient Bayesian inference algorithms. While ODE-based inference methods, such as generalized profiling and gradient matching, scale more easily to larger networks, they require the observation of transients for identifiability. In the low-copy, noise-dominated regimes typical of TF dynamics, the CLE provides a more informative generative model by exploiting stationary fluctuations which are not captured by deterministic models.

The CLE assumes a well-stirred, constant-volume system and does not provide a good approximation when molecule numbers approach zero [38]. Since the CLE likelihood is constructed from transition densities, residual transients from insufficient burn-in do not violate the model assumptions and are handled correctly. However, extrinsic sources of non-stationarity, such as cell-cycle effects or slow drifts in cellular state, are not captured by the intrinsic-noise CLE model. Depending on their timescale, such fluctuations could be misattributed to regulatory interactions, potentially generating spurious edges. Despite these limitations, our results demonstrate that a CLE-based inference approach can be used to successfully recover network topology and parameters from simulated gene expression time series across a range of biologically relevant regimes, including systems with cross-regulation and strong regulatory feedback. The proposed framework is a proof of concept demonstrated using small canonical network motifs, laying the foundation for future extensions to larger, more complex networks and applications to experimental data.

Central to the proposed Bayesian approach is the use of horseshoe priors [21], which reflect the assumed sparsity of interactions and allow for the accurate recovery of network architecture. We have shown that such priors suppress spurious edges while retaining true regulatory interactions. We have also tried alternatives, such as Laplacian priors [73] (not shown), and found that well-tuned horseshoe priors provided consistently sharper discrimination between existing and absent interactions.

We used a particular form of regulatory interactions to define the statistical model and the resulting likelihood functions, such as the AND-gate model of cross-regulation. Other regulatory logic functions can be incorporated in this framework, and our approach can be extended to determine which function best fits the data if multiple candidates are included in the model.

Inference in GRNs is subject to both structural non-identifiability, when the model equations do not uniquely determine certain parameters regardless of data quantity [62], and practical non-identifiability, when finite, noisy observations fail to constrain parameter estimates [74,75]. The notion of structural identifiability differs across levels of description. At the CME level, structural identifiability refers to whether parameters can be uniquely determined from the exact probability law p(X,t∣θ) [39,72]. At the CLE level, it refers to identifiability from the stationary path law of the SDE [62,74]. At the level of the Euler–Maruyama discretization of the CLE, it refers to whether parameters can be inferred using the Gaussian transition densities at the observed time points [40,46].

The maximal transcription rates ($\beta_{ij}$, $\gamma_{ij}$, $\eta_{i}$ and $\rho_{i}$) and degradation rates ($\alpha_{i}$) are identifiable from the stationary CLE likelihood *provided that the system operates in the transition regions of the different Hill functions*. Transcription rates enter the drift and diffusion terms as multiplicative scaling factors of the Hill functions, whereas degradation rates enter linearly in $X_{i}$, giving these two classes of parameters structurally distinct contributions to the mean and variance of the transition density. This allows them to be disentangled from the joint mean-variance structure of the likelihood. By contrast, the Hill parameters K and n modulate only the shape of the Hill function, and different (K,n) combinations can produce nearly identical Hill functions over the concentration range visited at stationarity, rendering them practically non-identifiable from stationary data alone [16,31,49,50]. Fixing these parameters to known values is therefore needed to overcome this structural limitation. Reliable inference of K and n requires informative priors or perturbation experiments spanning multiple operating regimes.

Identifiability can nevertheless break down in specific structural and practical settings, including Hill function saturation, near-linear operation and linear dependencies among AND-gate and single-input parameters in combinatorial models. Practical identifiability is further shaped by data characteristics, such as trajectory length and sampling interval Δt. Further analysis of these conditions is provided in S9 in the supplementary material. Non-isomorphic networks can produce indistinguishable stationary path statistics in two main settings. First, when the stationary distribution of a regulating TF lies in the saturated or near-linear regime of the Hill function. Second, self-activation and self-repression can be parametrized to produce identical marginal steady-state distributions. However, the CLE likelihood exploits the temporal autocorrelation structure of stationary fluctuations, which differs between these regulatory modes, enabling their discrimination. Inference methods based solely on marginal statistics cannot resolve this ambiguity.

Our approach has several limitations. First, computational and data requirements grow with network size: inference for N genes involves $O(N^{2})$ parameters, making inference in larger networks (>5 genes) challenging. In the present work, in each case, we repeated the inference procedure 50 times using 3000 measurements from each of 50 independent realizations. As shown in figure S12 in the supplementary material, posterior variance decreased monotonically with trajectory length. Inference in larger networks requires a larger number of observations, and a systematic investigation of how data requirements scale with network size is an important direction for future work. Second, for validation we used only synthetic data under idealized conditions. While this is a necessary first step in validating an inference method, we ignored extrinsic noise, measurement error and parameter non-stationarity present in real experiments [36]. While we have shown that the proposed framework works under idealized conditions, experimental validation using observations of synthetic gene circuits with known topology is an important next step. This will require extending the model to capture extrinsic noise, fluorescence measurement models and parameter non-stationarity. Third, our model assumes Hill function kinetics, CLE approximations valid at moderate copy numbers and negligible time delays—assumptions that can be violated in biological systems and may thus impact inference. Future work should address these limitations through benchmarking, experimental validation and model extensions that incorporate biologically important system properties.

Several avenues are available to address these challenges. First, incorporating both intrinsic and extrinsic noise sources into the likelihoods would yield more biologically realistic models. The likelihood is constructed directly from the discretized CLE, and the observed TF counts are assumed to be exact measurements of the true molecular state. We did not include observation noise which will be present in any realistic application. Incorporating measurement noise, for example, would transform the problem to inference from a partially observed diffusion process, requiring methods, such as particle MCMC or Kalman-type filtering [42,76]. We leave this extension to future work. Second, systematic validation on experimental time-series data will be essential to establish practical utility. Third, improving scalability through approximate inference schemes [76], dimensionality reduction [77] or variational Bayesian approaches [78] will broaden applicability to larger systems. Finally, extending the framework to more complex network architectures, including feedback loops and cross-regulatory modules, could help uncover design principles of natural gene networks at larger scales. Together, these directions can enhance both the computational feasibility and biological relevance of the method, paving the way for systems-level studies of gene regulation.

## Supplementary Material



## Data Availability

Code reproducing all results in the paper: https://github.com/gnashi21/Bayesian-Inference-of-Gene-Regulatory-Networks-at-Stochastic-Steady-State. Supplementary material is available online [79].
