# Supplementary material for: Bayesian inference of gene regulatory networks at stochastic steady state
Source: J R Soc Interface. 2026 Sep 9;23(242):20260040. doi: 10.1098/rsif.2026.0040 (PMC13561089; doi:10.1098/rsif.2026.0040)
Supplement: Supplementary file 1 [file rsif.2026.0040.zip › rsif20260040_si_001.pdf]

Bayesian Inference of Gene Regulatory Networks at Stochastic Steady State

Anshi Gupta[1], Ryeongkyung Yoon[3], and Krešimir Josić*[1,2,4]

[1]Department of Mathematics, University of Houston, Houston, TX 77204, USA
[2]Department of Biology and Biochemistry, University of Houston, Houston, TX 77204, USA
[3]Department of Mathematics and Computer Science, Wabash College, Crawfordsville, IN 47933, USA
[4]NSF-Simons National Institute for Theory and Mathematics in Biology, Chicago, IL 60611.

This section provides detailed descriptions of the synthetic datasets, likelihood formulations, and expanded inference results for each gene–regulatory motif described in the main text. For every motif, we include:

- **Data generation:** Synthetic trajectories generated using the stochastic simulation algorithm (SSA), together with the propensity functions and the true parameter values used in each simulation. These are summarized in tables specific to each motif.
- **Likelihood specification:** The drift and diffusion terms entering the Gaussian transition density obtained from the chemical Langevin equation (CLE).
- **Expanded inference results:** Posterior summaries (mean, standard deviation, and $\hat{R}$ statistics) for all inferred parameters, together with joint posterior distributions and box plots.

Each section below follows this structure consistently: (i) description of the synthetic data, (ii) explicit drift and diffusion terms for the likelihood, and (iii) expanded posterior results including all diagnostic figures and tables.

1 Notation Table

We summarize all notations and model parameters used in this paper in Table 1, and provide a computational summary of inference across all network motifs in Table 2.

Table 1: Key notation used in the Section 2

Symbol	Meaning
p	Number of genes/TFs in the network
$\mathbf{X}(t) \in \mathbb{R}^p$	State vector of TF concentrations
$\boldsymbol{\beta}, \boldsymbol{\gamma} \in \mathbb{R}^{p \times p}$	Activation and repression strength matrices
$\boldsymbol{\alpha} \in \mathbb{R}^{p \times p}$	Diagonal matrix of degradation rates
$\mathbf{A}(\mathbf{X}), \mathbf{R}(\mathbf{X}) \in \mathbb{R}^{p \times p}$	Matrix-valued Hill functions for activation/repression
q	Number of possible combinatorial input combinations
$\boldsymbol{\rho} \in \mathbb{R}^{p \times q}$	AND-gate regulatory strengths
$\boldsymbol{\Omega} \in \{0, 1\}^{p \times q}$	Binary indicators for AND gates
$\boldsymbol{\nu} \in \mathbb{R}^{p \times p}$	Single-reaction regulatory strengths
$\boldsymbol{\theta} \in \{0, 1\}^{p \times p}$	Binary indicators for activation vs. repression

*Corresponding author: josic@math.uh.edu

Table 2: Computational summary of inference across network motifs. Parameter count includes all continuous and discrete parameters estimated. Time series length is the number of measurements per realization. MCMC samples refers to the number of post-warmup HMC draws per chain. Runtime is per realization on a single CPU core.

Motif	Genes	Parameters	Time series	MCMC draws	Chains	Steps	Runtime
Self-regulation	1	3	3000	10,000 draw, 5000 tune	4	1	~2 min
Toggle switch	2	7	3000	10,000 draw, 5000 tune	4	1	~10 min
Toggle + AND gate	2	9 + 8 binary	3000	10,000 draw, 5000 tune	4	1	~25 min
Repressilator	3	12	3000	10,000 draw, 5000 tune	4	1	~45 min
C1-FFL	3	10 + 12 binary	3000	10,000 draw, 5000 tune	4	2	~2 hr

2 Single-Gene Motifs

2.1 Data

We generate synthetic time-series data using the stochastic simulation algorithm (SSA). The regulatory functions and parameter values used to simulate single-gene systems are summarised in Table 3. Parameters were chosen so that their marginal statistics remain nearly the same (See histograms shown in Fig. 1). We chose $\Delta t = 1$ to generate the synthetic trajectories.

As shown in Fig. 2, this sampling interval strikes a balance between temporal resolution and autocorrelation decay, and in our case was found to be the optimal choice. The same value of Δt was used to generate all synthetic datasets across the examples presented in this work.

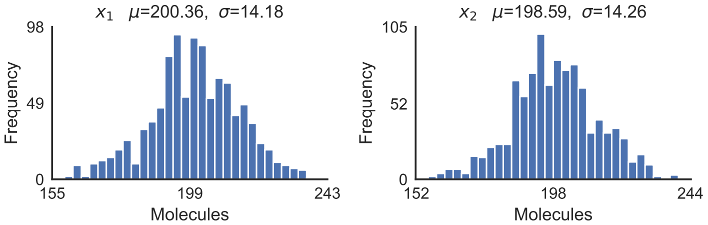

Figure 1: Histograms of the time series for the single gene self-activating (left) and self-repressing system (right). Both exhibit nearly identical marginal means and variances.

Table 3: Propensity functions and parameter values used to generate the SSA data for the single-gene motifs.

**Transcription Factor**	**Propensities**	$\boldsymbol{\beta/\gamma}$	$\boldsymbol{K}$	$\boldsymbol{n}$	$\boldsymbol{\alpha}$
$\boldsymbol{X_1}$	$f_a(X_1),\ \alpha_1 X_1$	10	29	4	0.05
$\boldsymbol{X_2}$	$f_r(X_2),\ \alpha_2 X_2$	10	500	4	0.05

2.2 Definition of likelihoods

The drift and diffusion terms used to define the likelihood in the single gene case are:

$$\begin{aligned} \mu_i &= \left(\frac{\beta_{ii}\, X_i^n}{X_i^n + K_i^n} + \frac{\gamma_{ii}\, K_i^n}{X_i^n + K_i^n} - \alpha_i\, X_i \right) \Delta t, \\ \sigma_i &= \left(\sqrt{\frac{\beta_{ii}\, X_i^n}{X_i^n + K_i^n} + \frac{\gamma_{ii}\, K_i^n}{X_i^n + K_i^n} + \alpha_i\, X_i} \right) \sqrt{\Delta t}. \end{aligned} \tag{1}$$

The likelihood for each species is then modeled using a normal transition density $N(\mu_i, \sigma_i)$ for $i = 1, 2$.

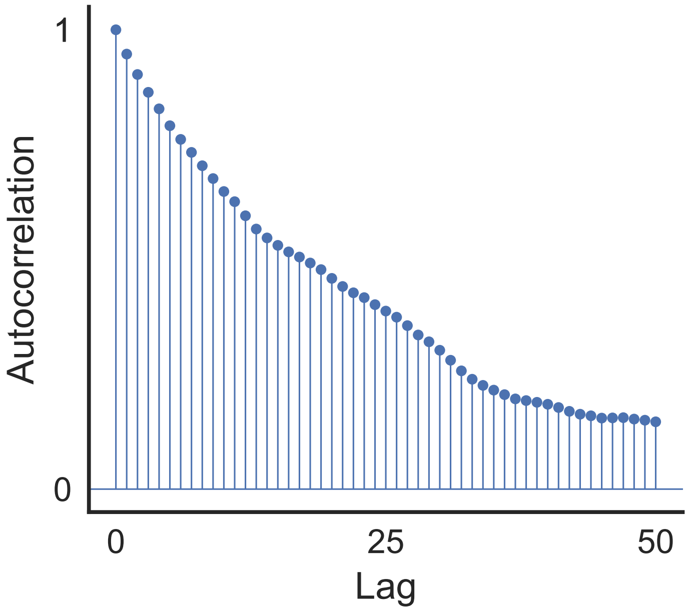

Figure 2: Autocorrelation function for TF X_1

2.3 Expanded Results for Inference

Table 4 reports the posterior summaries for all inferred parameters. Across all chains, the $\hat{R}$ values were equal to 1, indicating excellent convergence and well-mixed samples. The posterior means closely matched the true values used to generate the data, demonstrating accurate recovery of the model parameters. The joint posterior distributions, along with the marginal posterior distributions on the diagonal, are shown in Fig. 3.

Table 4: Posterior summary statistics for the inferred parameters in the single-gene motifs.

**Parameter**	**True**	**Mean**	**SD**	$\hat{R}$	**HDI 2.5%**	**HDI 97.5%**	**ESS Bulk**	**ESS Tail**
β_{11}	10.00	9.075	0.398	1.00	8.310	9.868	39 732.0	30 198.0
γ_{11}	0.00	0.001	0.467	1.00	−0.946	1.052	32 630.0	28 945.0
α_1	0.05	0.046	0.002	1.00	0.042	0.050	39 555.0	28 045.0
β_{22}	0.00	−0.003	0.463	1.00	−1.015	0.944	33 113.0	28 801.0
γ_{22}	10.00	9.564	0.424	1.00	8.739	10.402	40 617.0	30 248.0
α_2	0.05	0.048	0.002	1.00	0.044	0.053	40 638.0	27 255.0

2.4 Misidentification of edges in Saturated Regime

For the single-gene autoregulatory motif, when the stationary distribution of X lies entirely in the saturated regime of the Hill function, inference fails to correctly identify the regulatory interaction type. In this regime, activation and repression produce indistinguishable transition densities, leading to misidentification of the regulatory edge. The results are presented in the Table 5.This diagnostic illustrates the practical importance of ensuring that the stationary distribution of the regulating TF spans the transition region of the Hill function, as enforced in all examples presented in the main text.

Table 5: Posterior summary statistics for the saturated regime of the one-gene system.

Parameter	True	Mean	SD	HDI 3%	HDI 97%	ESS Bulk	ESS Tail	$\hat{R}$
β_{11}	10	4.773	7.507	−8.570	9.626	7.0	25.0	1.61
γ_{11}	0	254.288	440.338	−2.005	1 037.626	6.0	25.0	1.69
α_1	0.05	0.061	0.034	0.000	0.085	7.0	27.0	1.65

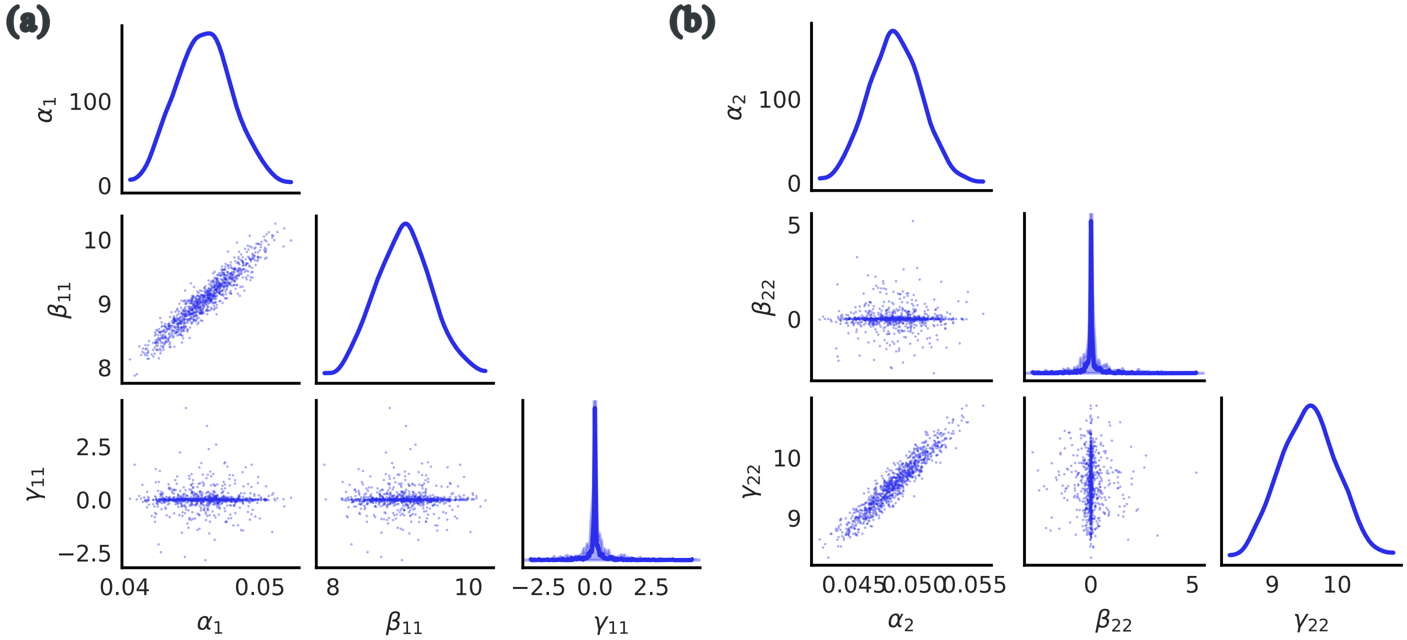

Figure 3: **Joint posterior distributions of inferred parameters for (a) self-activation and (b) self-repression motifs.**

3 Two-Gene Motifs

In this section, we present the data generation setup and expanded inference results for the two-gene motif with cross-regulation example introduced in the main text.

3.1 Data

Synthetic time-series data was generated using the SSA. The regulatory functions and parameter values used in the simulations are summarized in Table 6.

Table 6: Propensity functions and parameter values for X_1 and X_2 in the two-gene motifs.

Transcription Factor	Propensities	β/γ	K	n	α
X_1	$f_a(X_2),\ \alpha_1 X_1$	10	80	4	0.03
X_2	$f_r(X_1),\ \alpha_2 X_2$	20	50	4	0.02

3.2 Definition of likelihoods

The explicit expressions for the drift and diffusion terms for each gene X_i are given by:

$$\mu_1 = \left(\frac{\gamma_{11} K_1^{n_1}}{X_1^{n_1} + K_1^{n_1}} + \frac{\beta_{11} X_1^{n_1}}{X_1^{n_1} + K_1^{n_1}} + \frac{\gamma_{12} K_1^{n_1}}{X_2^{n_1} + K_1^{n_1}} + \frac{\beta_{12} X_2^{n_1}}{X_2^{n_1} + K_1^{n_1}} - \alpha_1 X_1 \right) \Delta t,$$
$$\sigma_1 = \sqrt{\frac{\gamma_{11} K_1^{n_1}}{X_1^{n_1} + K_1^{n_1}} + \frac{\beta_{11} X_1^{n_1}}{X_1^{n_1} + K_1^{n_1}} + \frac{\gamma_{12} K_1^{n_1}}{X_2^{n_1} + K_1^{n_1}} + \frac{\beta_{12} X_2^{n_1}}{X_2^{n_1} + K_1^{n_1}} + \alpha_1 X_1} \sqrt{\Delta t}. \tag{2}$$

$$\mu_2 = \left(\frac{\gamma_{22} K_2^{n_2}}{X_2^{n_2} + K_2^{n_2}} + \frac{\beta_{22} X_2^{n_2}}{X_2^{n_2} + K_2^{n_2}} + \frac{\gamma_{21} K_2^{n_2}}{X_1^{n_2} + K_2^{n_2}} + \frac{\beta_{21} X_1^{n_2}}{X_1^{n_2} + K_2^{n_2}} - \alpha_2 X_2 \right) \Delta t,$$
$$\sigma_2 = \sqrt{\frac{\gamma_{22} K_2^{n_2}}{X_2^{n_2} + K_2^{n_2}} + \frac{\beta_{22} X_2^{n_2}}{X_2^{n_2} + K_2^{n_2}} + \frac{\gamma_{21} K_2^{n_2}}{X_1^{n_2} + K_2^{n_2}} + \frac{\beta_{21} X_1^{n_2}}{X_1^{n_2} + K_2^{n_2}} + \alpha_2 X_2} \sqrt{\Delta t}. \tag{3}$$

3.3 Expanded Results

Fig. 4 shows box plots of all the inferred parameters, and Table 7 summarizes posterior means, standard deviations, and $\hat{R}$ values. Joint and marginal posterior distributions are shown in Fig. 5.

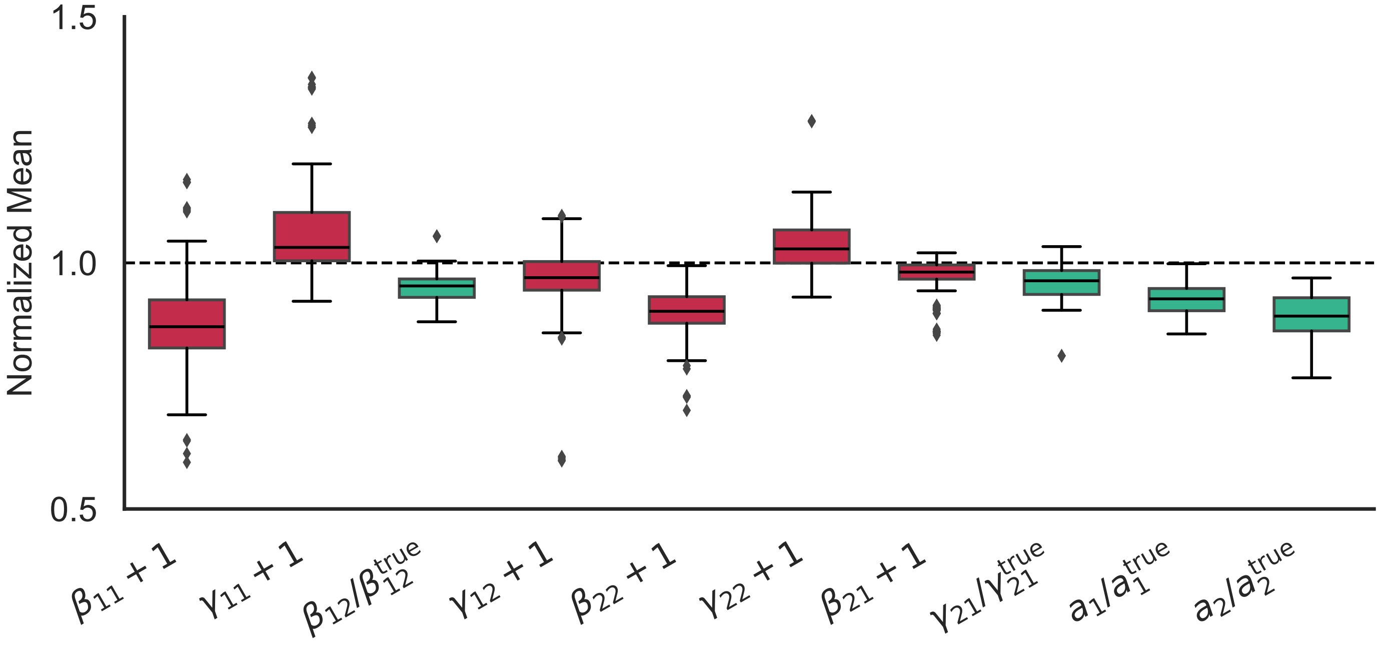

Figure 4: Box plots of all the inferred parameters for the two-gene motif.

4 Two-Gene Motifs with an AND Gate

In this section, we provide the details for the two-gene AND-gate example described in the main text.

4.1 Data

Synthetic trajectories were generated using the SSA with the propensity functions and parameter values listed in Table 8.

Table 7: Posterior summaries for selected inferred parameters in the two-gene motif.

**Parameter**	**True**	**Mean**	**SD**	**HDI 3%**	**HDI 97%**	**ESS Bulk**	**ESS Tail**	$\hat{R}$
β_{11}	0	−0.043	0.282	−0.576	0.461	8 760.0	8 361.0	1.0
β_{12}	10	9.185	0.537	8.145	10.159	14 884.0	14 134.0	1.0
β_{21}	0	−0.023	0.092	−0.216	0.131	17 950.0	21 410.0	1.0
β_{22}	0	−0.092	0.245	−0.608	0.190	6 639.0	3 285.0	1.0
γ_{11}	0	0.103	0.360	−0.483	0.999	18 077.0	12 534.0	1.0
γ_{12}	0	−0.036	0.243	−0.522	0.411	11 306.0	12 196.0	1.0
γ_{21}	20	20.212	1.121	18.080	22.330	34 558.0	33 427.0	1.0
γ_{22}	0	0.012	0.109	−0.202	0.242	16 101.0	10 732.0	1.0
α_1	0.03	0.027	0.003	0.022	0.031	10 213.0	6 384.0	1.0
α_2	0.02	0.018	0.003	0.013	0.022	6 374.0	3 098.0	1.0

Table 8: Propensity functions and parameter values for X_1 and X_2 in the two-gene motif with an AND gate.

**Transcription Factor**	**Propensities**	$\beta/\gamma/\eta$	K	n	α
X_1	$H_{rr}(X_2(t)),\ \alpha_1 X_1(t)$	20	100, 80	2	0.06
X_2	$f_a(X_1(t)),\ \alpha_2 X_2(t)$	10	70	2	0.055

4.2 Definition of likelihoods

The drift and diffusion terms for each TF are given by:

$$
\begin{aligned}
\mu_1 &= \Delta t\Bigg(\rho_1\left[\frac{\omega_1\omega_2\,X_1^{n_1}X_2^{n_2}+\omega_1\tilde{\omega}_2\,X_1^{n_1}K_2^{n_2}+\tilde{\omega}_1\omega_2\,K_1^{n_1}X_2^{n_2}+\tilde{\omega}_1\tilde{\omega}_2\,K_1^{n_1}K_2^{n_2}}{(K_1^{n_1}+X_1^{n_1})(K_2^{n_2}+X_2^{n_2})}\right] \\
&\quad+\nu_{11}\frac{\theta_{11}X_1^{n_1}+\tilde{\theta}_{11}K_1^{n_1}}{X_1^{n_1}+K_1^{n_1}}+\nu_{12}\frac{\theta_{12}X_2^{n_2}+\tilde{\theta}_{12}K_2^{n_2}}{X_2^{n_2}+K_2^{n_2}}-\alpha_1 X_1\Bigg) \\
\sigma_1(t) &= \sqrt{\Delta t}\Bigg(\sqrt{\rho_1\frac{\omega_1\omega_2\,X_1^{n_1}X_2^{n_2}+\omega_1\tilde{\omega}_2\,X_1^{n_1}K_2^{n_2}+\tilde{\omega}_1\omega_2\,K_1^{n_1}X_2^{n_2}+\tilde{\omega}_1\tilde{\omega}_2\,K_1^{n_1}K_2^{n_2}}{(K_1^{n_1}+X_1^{n_1})(K_2^{n_2}+X_2^{n_2})}} \\
&\quad+\beta_{11}\frac{\theta_{11}X_1^{n_1}+\tilde{\theta}_{11}K_1^{n_1}}{X_1^{n_1}+K_1^{n_1}}+\beta_{12}\frac{\theta_{12}X_2^{n_2}+\tilde{\theta}_{12}K_2^{n_2}}{X_2^{n_2}+K_2^{n_2}}+\alpha_1 X_1\Bigg)
\end{aligned} \tag{4}
$$

$$
\begin{aligned}
\mu_2 &= \Delta t\Bigg(\rho_2\frac{\omega_3\omega_4\,X_1^{n_3}X_2^{n_2}+\omega_3\tilde{\omega}_4\,X_1^{n_3}K_2^{n_2}+\tilde{\omega}_3\omega_4\,K_3^{n_3}X_2^{n_2}+\tilde{\omega}_3\tilde{\omega}_4\,K_3^{n_3}K_2^{n_2}}{(K_3^{n_3}+X_1^{n_3})(K_2^{n_2}+X_2^{n_2})} \\
&\quad+\nu_{21}\frac{\theta_{21}X_1^{n_3}+\tilde{\theta}_{21}K_3^{n_3}}{X_1^{n_3}+K_3^{n_3}}+\nu_{22}\frac{\theta_{22}X_2^{n_3}+\tilde{\theta}_{22}K_3^{n_3}}{X_2^{n_3}+K_3^{n_3}}-\alpha_2 X_2\Bigg) \\
\sigma_2(t) &= \sqrt{\Delta t}\Bigg(\sqrt{\rho_2\frac{\omega_3\omega_4\,X_1^{n_3}X_2^{n_2}+\omega_3\tilde{\omega}_4\,X_1^{n_3}K_2^{n_2}+\tilde{\omega}_3\omega_4\,K_3^{n_3}X_2^{n_2}+\tilde{\omega}_3\tilde{\omega}_4\,K_3^{n_3}K_2^{n_2}}{(K_3^{n_3}+X_1^{n_3})(K_2^{n_2}+X_2^{n_2})}} \\
&\quad+\sqrt{\nu_{21}\frac{\theta_{21}X_1^{n_3}+\tilde{\theta}_{21}K_3^{n_3}}{X_1^{n_3}+K_3^{n_3}}+\nu_{22}\frac{\theta_{22}X_2^{n_3}+\tilde{\theta}_{22}K_3^{n_3}}{X_2^{n_3}+K_3^{n_3}}+\alpha_2 X_2}\Bigg)
\end{aligned} \tag{5}
$$

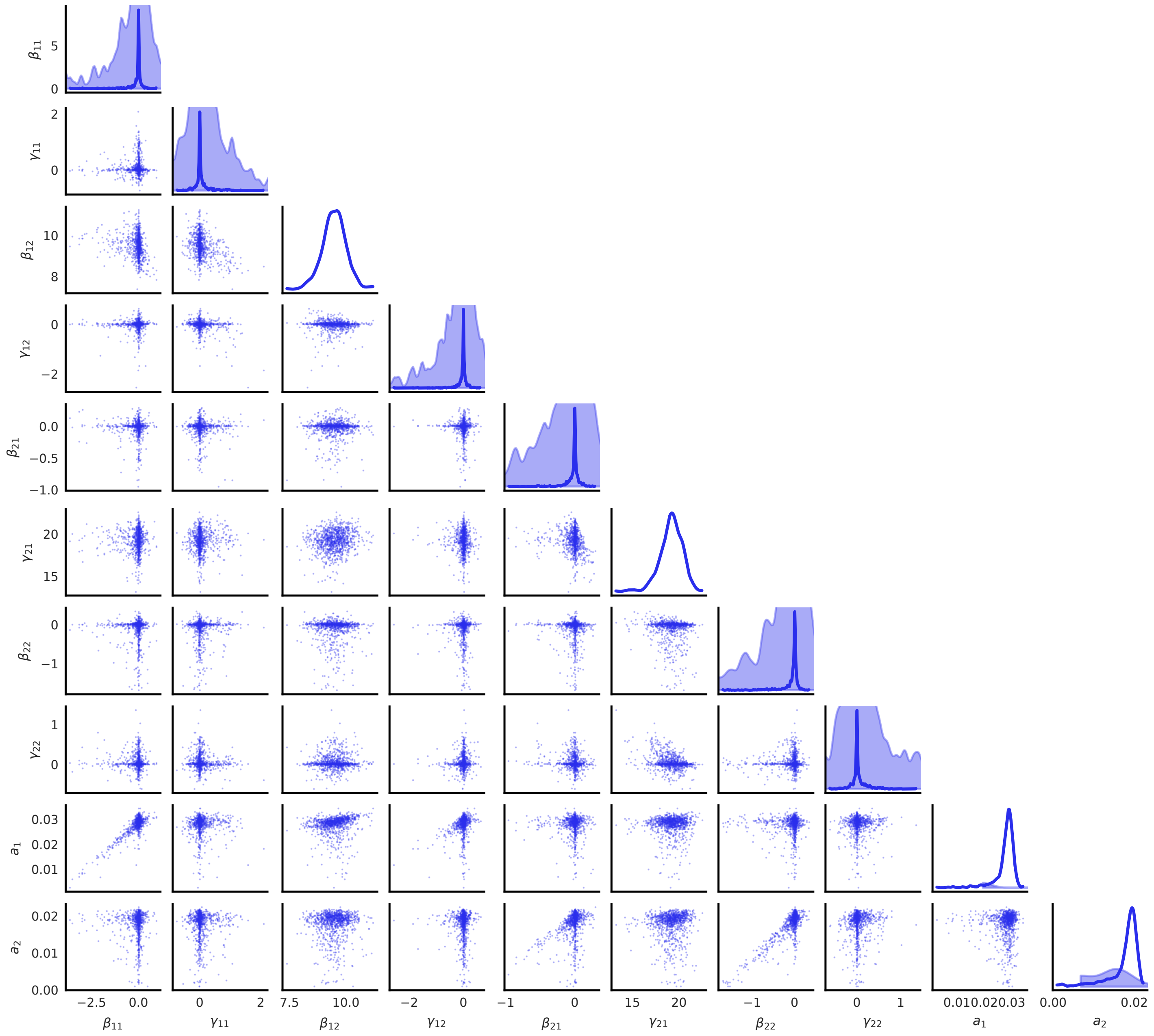

Figure 5: Joint posterior distributions of inferred parameters for the two-gene motif.

4.3 Expanded Results

Table 9 summarizes the inferred parameters. All $\hat{R}$ values are approximately 1, indicating good convergence, and the posterior means are close to the true generating values. The joint posterior densities with diagonal marginals are shown in Fig. 6.

5 Repressilator

In this section, we provide the full details for the three-gene repressilator example discussed in the main text.

Table 9: Summary statistics for inferred parameters for 2 gene AND gate

Parameter	True	Mean	SD	HDI 3%	HDI 97%	ESS Bulk	ESS Tail	$\hat{R}$
ν_{11}	0	−0.043	0.360	−0.614	0.481	7 407.615	3 631.150	1.001
ν_{12}	0	0.088	0.511	−0.760	1.024	10 778.498	10 479.044	1.000
ν_{21}	10	9.415	0.355	8.817	10.033	21 980.538	17 078.160	1.000
ν_{22}	0	0.030	0.386	−0.651	0.776	8 447.949	4 328.062	1.000
α_1	0.06	0.055	0.002	0.051	0.058	8 271.818	3 637.799	1.000
α_2	0.055	0.052	0.002	0.049	0.055	7 494.173	3 061.509	1.001
ρ_1	20	18.271	0.918	16.811	19.726	18 868.047	14 388.524	1.000
ρ_2	0	0.024	0.487	−0.839	0.935	15 080.334	11 210.560	1.000

5.1 Data

Synthetic trajectories are generated using the stochastic simulation algorithm (SSA). The propensity functions and the parameter values used in the simulations for the three proteins are listed in Table 10.

Table 10: Propensity functions and parameter values for X_1, X_2, and X_3 in the repressilator motif.

Transcription Factor	Propensities	β/γ	K	n	α
X_1	$f_r(X_3(t)),\ \alpha_1 X_1(t)$	10	90	2	0.03
X_2	$f_r(X_1(t)),\ \alpha_2 X_2(t)$	20	70	2	0.05
X_3	$f_r(X_2(t)),\ \alpha_3 X_3(t)$	15	50	2	0.04

5.2 Definition of likelihoods

The drift and diffusion terms for each species follow directly from the general CLE-based formulation. For each $i \in \{1, 2, 3\}$,

$$
\begin{aligned}
\mu_1(t) = \Delta t \Bigg(& \beta_{11} \frac{X_1^{n_1}}{K_1^{n_1} + X_1^{n_1}} + \gamma_{11} \frac{K_1^{n_1}}{K_1^{n_1} + X_1^{n_1}} + \beta_{12} \frac{X_2^{n_2}}{K_2^{n_2} + X_2^{n_2}} + \gamma_{12} \frac{K_2^{n_2}}{K_2^{n_2} + X_2^{n_2}} \\
& + \beta_{13} \frac{X_3^{n_3}}{K_3^{n_3} + X_3^{n_3}} + \gamma_{13} \frac{K_3^{n_3}}{K_3^{n_3} + X_3^{n_3}} - \alpha_1 X_1 \Bigg) \\
\sigma_1(t) = \sqrt{\Delta t} \Bigg(& \sqrt{\beta_{11} \frac{X_1^{n_1}}{K_1^{n_1} + X_1^{n_1}} + \gamma_{11} \frac{K_1^{n_1}}{K_1^{n_1} + X_1^{n_1}} + \beta_{12} \frac{X_2^{n_2}}{K_2^{n_2} + X_2^{n_2}}} \\
& \sqrt{+\gamma_{12} \frac{K_2^{n_2}}{K_2^{n_2} + X_2^{n_2}} + \beta_{13} \frac{X_3^{n_3}}{K_3^{n_3} + X_3^{n_3}} + \gamma_{13} \frac{K_3^{n_3}}{K_3^{n_3} + X_3^{n_3}} + \alpha_1 X_1} \Bigg)
\end{aligned}
\tag{6}
$$

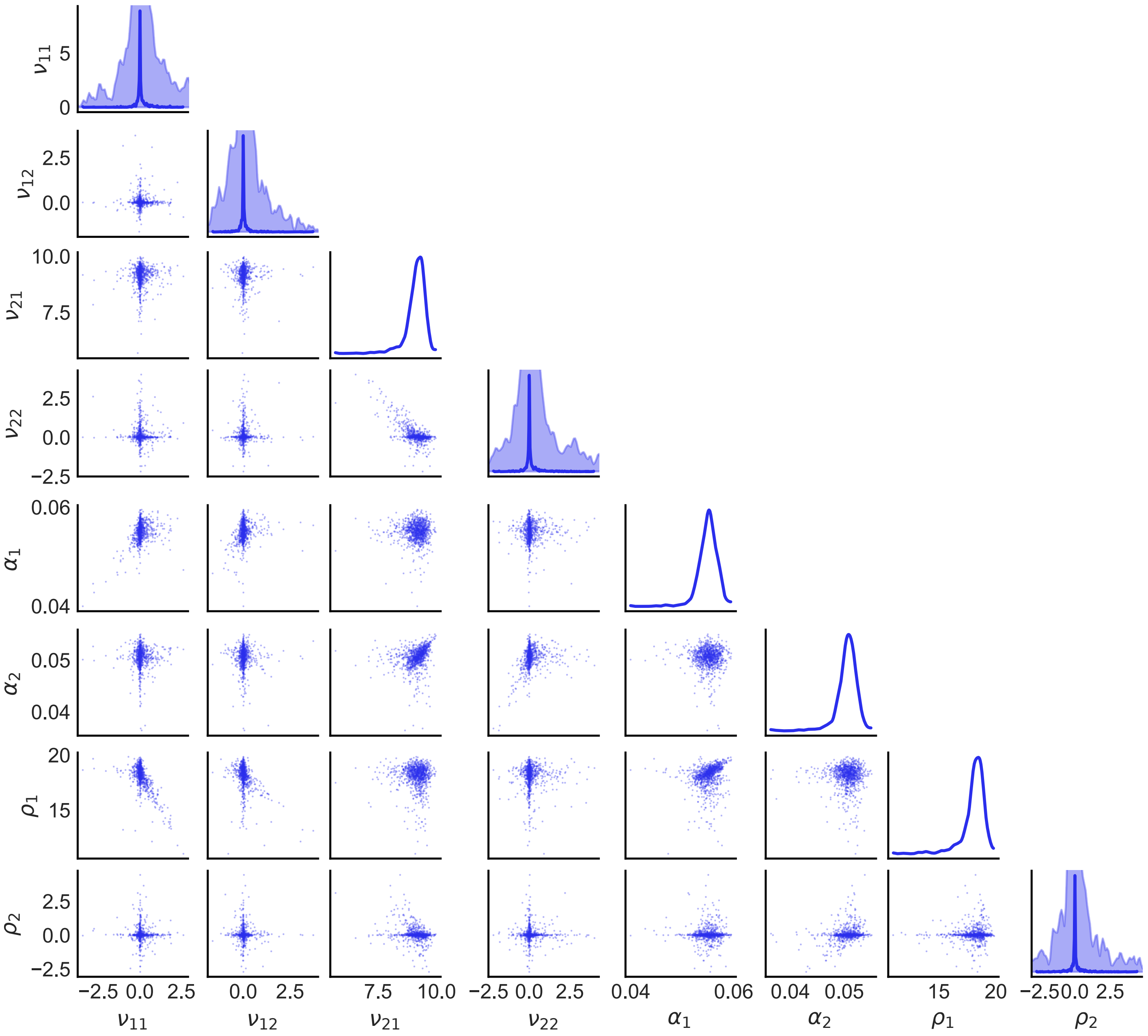

Figure 6: Joint posterior distributions for the two-gene AND-gate example.

$$
\begin{aligned}
\mu_2(t) &= \Delta t \Bigg(\beta_{22} \frac{X_2^{n_2}}{K_2^{n_2} + X_2^{n_2}} + \gamma_{22} \frac{K_2^{n_2}}{K_2^{n_2} + X_2^{n_2}} + \beta_{21} \frac{X_1(t)^{n_1}}{K_1^{n_1} + X_1^{n_1}} + \gamma_{21} \frac{K_1^{n_1}}{K_1^{n_1} + X_1^{n_1}} \\
&\quad + \beta_{23} \frac{X_3^{n_3}}{K_3^{n_3} + X_3^{n_3}} + \gamma_{23} \frac{K_3^{n_3}}{K_3^{n_3} + X_3^{n_3}} - \alpha_2 X_2 \Bigg) \\
\sigma_2(t) &= \sqrt{\Delta t} \sqrt{\beta_{22} \frac{X_2^{n_2}}{K_2^{n_2} + X_2^{n_2}} + \gamma_{22} \frac{K_2^{n_2}}{K_2^{n_2} + X_2^{n_2}} + \beta_{21} \frac{X_1^{n_1}}{K_1^{n_1} + X_1^{n_1}} + \gamma_{21} \frac{K_1^{n_1}}{K_1^{n_1} + X_1^{n_1}}} \\
&\quad \sqrt{+ \beta_{23} \frac{X_3^{n_3}}{K_3^{n_3} + X_3^{n_3}} + \gamma_{23} \frac{K_3^{n_3}}{K_3^{n_3} + X_3^{n_3}} + \alpha_2 X_2}
\end{aligned}
\tag{7}
$$

$$\mu_3(t) = \Delta t \Bigg(\beta_{31} \frac{X_1^{n_1}}{K_1^{n_1} + X_1^{n_1}} + \gamma_{31} \frac{K_1^{n_1}}{K_1^{n_1} + X_1^{n_1}} + \beta_{32} \frac{X_2^{n_2}}{K_2^{n_2} + X_2^{n_2}} + \gamma_{32} \frac{K_2^{n_2}}{K_2^{n_2} + X_2^{n_2}} + \beta_{33} \frac{X_3^{n_3}}{K_3^{n_3} + X_3^{n_3}} + \gamma_{33} \frac{K_3^{n_3}}{K_3^{n_3} + X_3^{n_3}} - \alpha_3 X_3 \Bigg)$$

$$\sigma_3(t) = \sqrt{\Delta t} \sqrt{\beta_{31} \frac{X_1^{n_1}}{K_1^{n_1} + X_1^{n_1}} + \gamma_{31} \frac{K_1^{n_1}}{K_1^{n_1} + X_1^{n_1}} + \beta_{32} \frac{X_2^{n_2}}{K_2^{n_2} + X_2^{n_2}} + \gamma_{32} \frac{K_2^{n_2}}{K_2^{n_2} + X_2^{n_2}} + \beta_{33} \frac{X_3^{n_3}}{K_3^{n_3} + X_3^{n_3}} + \gamma_{33} \frac{K_3^{n_3}}{K_3^{n_3} + X_3^{n_3}} + \alpha_3 X_3} \tag{8}$$

5.3 Expanded Results

Table 11 reports posterior summary statistics for all inferred parameters. All chains show excellent convergence ($\hat{R} \approx 1$), and the posterior means closely match the true values, indicating accurate parameter recovery. Box plots and joint posterior distributions are shown in Fig. 7 and 8, respectively.

Table 11: Posterior summary statistics with true parameter values for the Repressilator.

Parameter	True	Mean	SD	HDI 3%	HDI 97%	ESS Bulk	ESS Tail	$\hat{R}$
β_{11}	0	0.003	0.218	−0.387	0.483	21 008.274	13 103.475	1.00
β_{12}	0	0.114	0.279	−0.278	0.768	31 491.663	32 838.053	1.00
β_{13}	0	0.016	0.160	−0.274	0.377	34 019.094	27 931.098	1.00
β_{21}	0	0.013	0.127	−0.213	0.309	39 923.452	31 940.460	1.00
β_{22}	0	−0.007	0.211	−0.418	0.422	18 762.226	11 417.301	1.00
β_{23}	0	0.087	0.230	−0.230	0.622	35 973.841	35 182.683	1.00
β_{31}	0	0.010	0.147	−0.284	0.328	39 040.920	31 534.366	1.00
β_{32}	0	−0.017	0.135	−0.303	0.246	35 896.806	27 380.002	1.00
β_{33}	0	−0.042	0.287	−0.533	0.469	17 597.633	10 408.208	1.00
γ_{11}	0	0.125	0.417	−0.475	1.100	34 218.903	34 282.216	1.00
γ_{12}	0	−0.052	0.290	−0.677	0.429	29 911.045	21 887.815	1.00
γ_{13}	10	9.485	0.536	8.428	10.360	38 330.922	36 150.975	1.00
γ_{21}	20	18.287	0.909	16.530	19.864	41 935.872	37 785.044	1.00
γ_{22}	0	0.048	0.203	−0.290	0.498	42 314.512	36 592.866	1.00
γ_{23}	0	−0.032	0.195	−0.468	0.305	32 376.890	24 716.220	1.00
γ_{31}	0	−0.049	0.353	−0.741	0.601	36 800.349	27 400.775	1.00
γ_{32}	15	14.197	0.524	13.200	15.149	40 585.913	37 586.908	1.00
γ_{33}	0	0.011	0.158	−0.295	0.352	42 725.081	36 518.755	1.00
α_1	0.03	0.030	0.001	0.027	0.032	19 980.802	10 277.720	1.00
α_2	0.05	0.046	0.002	0.042	0.050	19 017.458	9 054.320	1.00
α_3	0.04	0.038	0.002	0.034	0.041	17 333.434	8 821.772	1.00

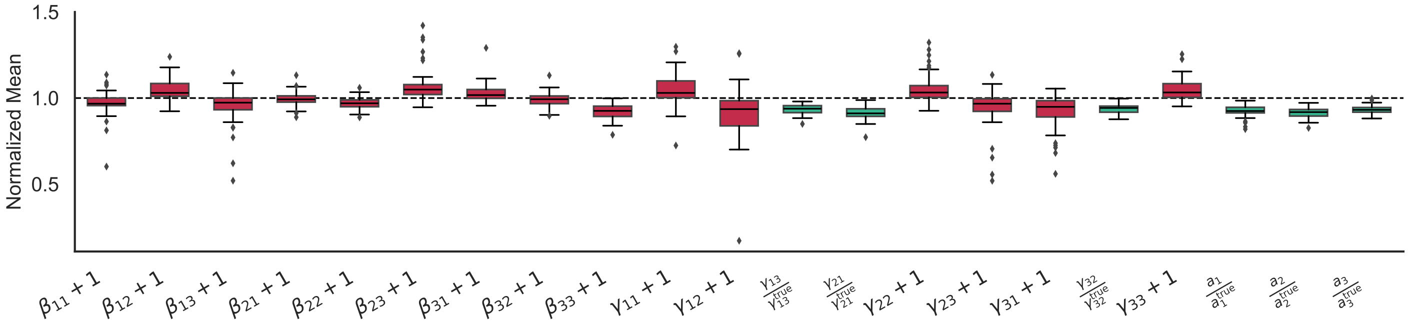

Figure 7: Box plots of posterior parameter means for the Repressilator.

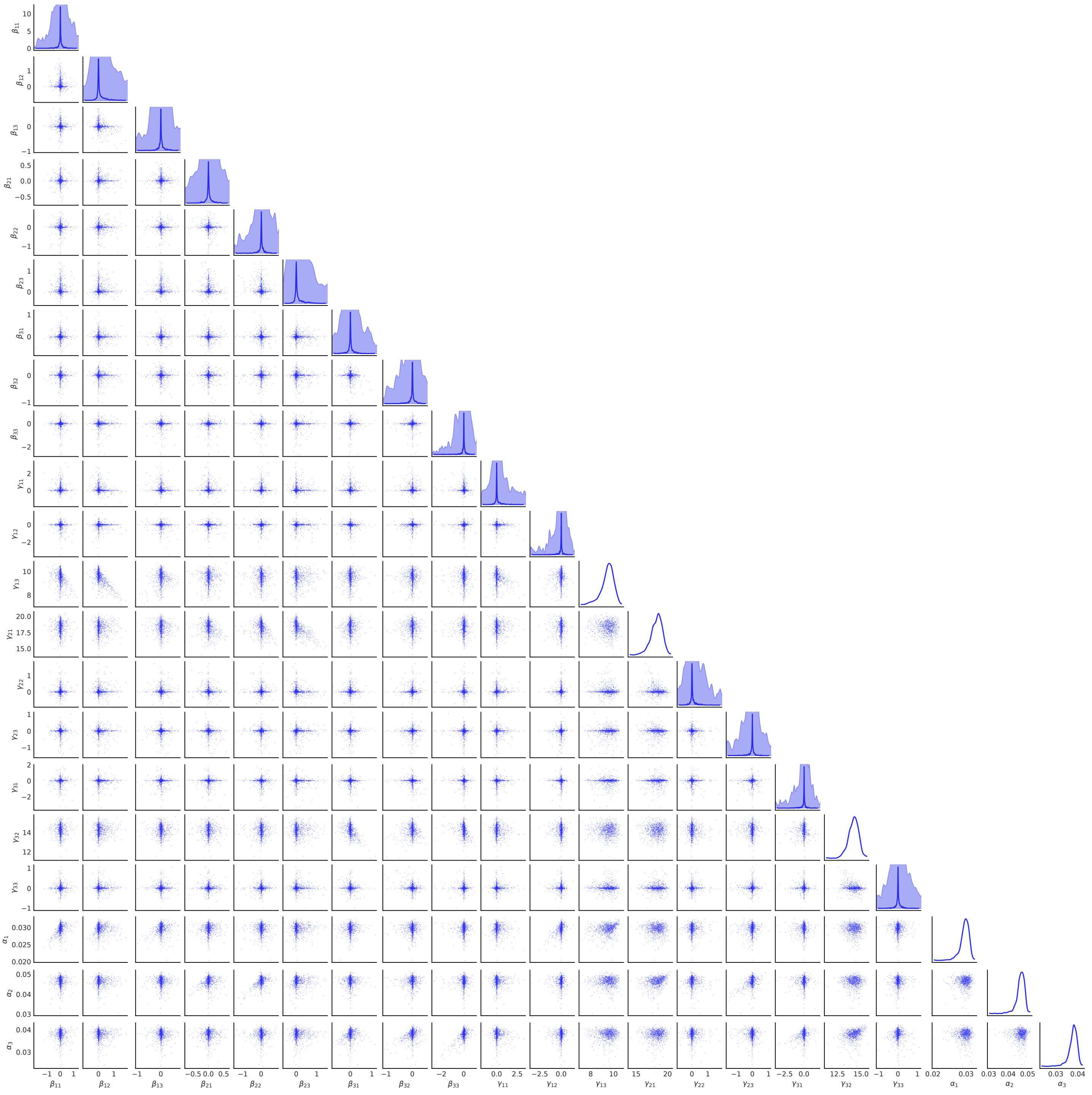

Figure 8: Joint posterior distributions for the Repressilator parameters.

6 Coherent Type 1 Motif

We now provide details for the Coherent Type 1 feed-forward loop discussed in the main text. This motif consists of a single regulatory interaction from X_1 to X_2, and an AND-gate at X_3 driven jointly by X_1 and X_2. Inference is performed in two steps: in Step 1, we infer the network topology using the model in Eq. (9); in Step 2, we use the inferred topology as prior structural knowledge and estimate all kinetic parameters using the full model in Eq. (8).

6.1 Data

Table 12 reports the propensity functions and parameter values used to generate the SSA data.

Table 12: Propensity functions and parameter values for X_1, X_2, and X_3 in the Coherent Type 1 motif.

**Transcription Factor**	**Propensities**	$\beta/\gamma/\eta$	K	n	α
X_1	$k = 5,\ \alpha_1 X_1$	–	–	3	0.06
X_2	$f_a(X_1),\ \alpha_2 X_2$	20	150	2	0.045
X_3	$H_{aa}(X_1, X_2),\ \alpha_3 X_3$	20	10, 50	3	0.05

6.2 Definition of likelihoods

Inference proceeds in two steps. First, we provide the drift and diffusion terms used in Step 1 for topology inference, followed by the corresponding expressions used in Step 2 for parameter estimation.

6.2.1 Step 1

$$
\begin{aligned}
\mu_1 = \Delta t \Bigg(& \nu_{12}\left[\theta_{12}\frac{X_2^n}{X_2^n+K^n} + \hat{\theta}_{12}\frac{K^n}{X_2^n+K^n}\right] + \nu_{13}\left[\theta_{13}\frac{X_3^{n_1}}{X_3^{n_1}+K_1^{n_1}} + \hat{\theta}_{13}\frac{K_1^{n_1}}{X_3^{n_1}+K_1^{n_1}}\right] \\
& + \rho_1\Bigg[\omega_{12}\omega_{13}\frac{X_2^n X_3^{n_1}}{(X_2^n+K^n)(X_3^{n_1}+K_1^{n_1})} + \hat{\omega}_{12}\omega_{13}\frac{K^n X_3^{n_1}}{(X_2^n+K^n)(X_3^{n_1}+K_1^{n_1})} \\
& \quad + \omega_{12}\hat{\omega}_{13}\frac{X_2^n K_1^{n_1}}{(X_2^n+K^n)(X_3^{n_1}+K_1^{n_1})} + \hat{\omega}_{12}\hat{\omega}_{13}\frac{K^n K_1^{n_1}}{(X_2^n+K^n)(X_3^{n_1}+K_1^{n_1})}\Bigg] + r_1 - \alpha_1 X_1 \Bigg) \\
\sigma_1 = \sqrt{\Delta t}\, & \sqrt{\nu_{12}\left[\theta_{12}\frac{X_2^n}{X_2^n+K^n} + \hat{\theta}_{12}\frac{K^n}{X_2^n+K^n}\right] + \nu_{13}\left[\theta_{13}\frac{X_3^{n_1}}{X_3^{n_1}+K_1^{n_1}} + \hat{\theta}_{13}\frac{K_1^{n_1}}{X_3^{n_1}+K_1^{n_1}}\right]} \\
& + \sqrt{\rho_1\Bigg[\omega_{12}\omega_{13}\frac{X_2^n X_3^{n_1}}{(X_2^n+K^n)(X_3^{n_1}+K_1^{n_1})} + \hat{\omega}_{12}\omega_{13}\frac{K^n X_3^{n_1}}{(X_2^n+K^n)(X_3^{n_1}+K_1^{n_1})}} \\
& \quad + \sqrt{\omega_{12}\hat{\omega}_{13}\frac{X_2^n K_1^{n_1}}{(X_2^n+K^n)(X_3^{n_1}+K_1^{n_1})} + \hat{\omega}_{12}\hat{\omega}_{13}\frac{K^n K_1^{n_1}}{(X_2^n+K^n)(X_3^{n_1}+K_1^{n_1})}\Bigg] + r_1 + \alpha_1 X_1}
\end{aligned}
\tag{9}
$$

$$\mu_2(t) = \Delta t \Bigg(\nu_{21} \left[\theta_{21} \frac{X_1^n}{X_1^n + K^n} + \hat{\theta}_{21} \frac{K^n}{X_1^n + K^n} \right] + \nu_{23} \left[\theta_{23} \frac{X_3^{n_2}}{X_3^{n_2} + K_2^{n_2}} + \hat{\theta}_{23} \frac{K_2^{n_2}}{X_3^{n_2} + K_2^{n_2}} \right]$$

$$+ \rho_2 \Bigg[\omega_{21}\omega_{23} \frac{X_1^n X_3^{n_2}}{(X_1^n + K^n)(X_3^{n_2} + K_2^{n_2})} + \hat{\omega}_{21}\omega_{23} \frac{K^n X_3^{n_2}}{(X_1^n + K^n)(X_3^{n_2} + K_2^{n_2})}$$
$$+ \omega_{21}\hat{\omega}_{23} \frac{X_1^n K_2^{n_2}}{(X_1^n + K^n)(X_3^{n_2} + K_2^{n_2})} + \hat{\omega}_{21}\hat{\omega}_{23} \frac{K^n K_2^{n_2}}{(X_1^n + K^n)(X_3^{n_2} + K_2^{n_2})} \Bigg] + r_2 - \alpha_2 X_2 \Bigg)$$

$$\sigma_2(t) = \sqrt{\Delta t} \sqrt{\nu_{21} \left[\theta_{21} \frac{X_1^n}{X_1^n + K^n} + \hat{\theta}_{21} \frac{K^n}{X_1^n + K^n} \right] + \nu_{23} \left[\theta_{23} \frac{X_3^{n_2}}{X_3^{n_2} + K_2^{n_2}} + \hat{\theta}_{23} \frac{K_2^{n_2}}{X_3^{n_2} + K_2^{n_2}} \right]} \tag{10}$$

$$\sqrt{+\rho_2 \Bigg[\omega_{21}\omega_{23} \frac{X_1^n X_3^{n_2}}{(X_1^n + K^n)(X_3^{n_2} + K_2^{n_2})} + \hat{\omega}_{21}\omega_{23} \frac{K^n X_3^{n_2}}{(X_1^n + K^n)(X_3^{n_2} + K_2^{n_2})}}$$
$$\sqrt{+\omega_{21}\hat{\omega}_{23} \frac{X_1^n K_2^{n_2}}{(X_1^n + K^n)(X_3^{n_2} + K_2^{n_2})} + \hat{\omega}_{21}\hat{\omega}_{23} \frac{K^n K_2^{n_2}}{(X_1^n + K^n)(X_3^{n_2} + K_2^{n_2})} \Bigg] + \alpha_2 X_2 + r_2}$$

$$\mu_3 = \Delta t \Bigg(\nu_{31} \frac{\theta_{31} X_1^{n_1} + \hat{\theta}_{31} K_1^{n_1}}{X_1^{n_1} + K_1^{n_1}} + \nu_{32} \frac{\theta_{32} X_2^{n_2} + \hat{\theta}_{32} K_2^{n_2}}{X_2^{n_2} + K_2^{n_2}}$$

$$+ \rho_3 \Bigg[\omega_{31}\omega_{32} \frac{X_1^{n_1} X_2^{n_2}}{(X_1^{n_1} + K_1^{n_1})(X_2^{n_2} + K_2^{n_2})} + \hat{\omega}_{31}\omega_{32} \frac{K_1^{n_1} X_2^{n_2}}{(X_1^{n_1} + K_1^{n_1})(X_2^{n_2} + K_2^{n_2})}$$
$$+ \omega_{31}\hat{\omega}_{32} \frac{X_1^{n_1} K_2^{n_2}}{(X_1^{n_1} + K_1^{n_1})(X_2^{n_2} + K_2^{n_2})} + \hat{\omega}_{31}\hat{\omega}_{32} \frac{K_1^{n_1} K_2^{n_2}}{(X_1^{n_1} + K_1^{n_1})(X_2^{n_2} + K_2^{n_2})} \Bigg] + r_3 - \alpha_3 X_3 \Bigg)$$

$$\sigma_3 = \sqrt{\Delta t} \sqrt{\nu_{31} \frac{\theta_{31} X_1^{n_1} + \hat{\theta}_{31} K_1^{n_1}}{X_1^{n_1} + K_1^{n_1}} + \nu_{32} \frac{\theta_{32} X_2^{n_2} + \hat{\theta}_{32} K_2^{n_2}}{X_2^{n_2} + K_2^{n_2}}} \tag{11}$$

$$\sqrt{+\rho_3 \Bigg[\omega_{31}\omega_{32} \frac{X_1^{n_1} X_2^{n_2}}{(X_1^{n_1} + K_1^{n_1})(X_2^{n_2} + K_2^{n_2})} + \hat{\omega}_{31}\omega_{32} \frac{K_1^{n_1} X_2^{n_2}}{(X_1^{n_1} + K_1^{n_1})(X_2^{n_2} + K_2^{n_2})}}$$
$$\sqrt{+\omega_{31}\hat{\omega}_{32} \frac{X_1^{n_1} K_2^{n_2}}{(X_1^{n_1} + K_1^{n_1})(X_2^{n_2} + K_2^{n_2})} + \hat{\omega}_{31}\hat{\omega}_{32} \frac{K_1^{n_1} K_2^{n_2}}{(X_1^{n_1} + K_1^{n_1})(X_2^{n_2} + K_2^{n_2})} \Bigg] + \alpha_3 X_3 + r_3}$$

Step2

$$\mu_1 = \Delta t \left(r_1 - \alpha_1 X_1 \right)$$
$$\sigma_1 = \sqrt{\Delta t} \sqrt{r_1 + \alpha_1 X_1} \tag{12}$$

$$\mu_2 = \Delta t\left(\beta_{21}\frac{X_1^n}{X_1^n + K^n} + \gamma_{21}\frac{K^n}{X_1^n + K^n} + \beta_{23}\frac{X_3^{n_2}}{X_3^{n_2} + K_2^{n_2}} + \gamma_{23}\frac{K_2^{n_2}}{X_3^{n_2} + K_2^{n_2}} + r_2 - \alpha_2 X_2\right)$$

$$\sigma_2 = \sqrt{\Delta t}\sqrt{\beta_{21}\frac{X_1^n}{X_1^n + K^n} + \gamma_{21}\frac{K^n}{X_1^n + K^n} + \beta_{23}\frac{X_3^{n_2}}{X_3^{n_2} + K_2^{n_2}} + \gamma_{23}\frac{K_2^{n_2}}{X_3^{n_2} + K_2^{n_2}} + \alpha_2 X_2 + r_2} \tag{13}$$

$$\mu_3 = \Delta t\left(\eta_1\frac{X_1^{n_1}X_2^{n_2}}{(X_1^{n_1} + K_1^{n_1})(X_2^{n_2} + K_2^{n_2})} + \eta_2\frac{K_1^{n_1}X_2^{n_2}}{(X_1^{n_1} + K_1^{n_1})(X_2^{n_2} + K_2^{n_2})}\right.$$
$$\left. + \eta_3\frac{X_1^{n_1}K_2^{n_2}}{(X_1^{n_1} + K_1^{n_1})(X_2^{n_2} + K_2^{n_2})} + \eta_4\frac{K_1^{n_1}K_2^{n_2}}{(X_1^{n_1} + K_1^{n_1})(X_2^{n_2} + K_2^{n_2})} + r_3 - \alpha_3 X_3\right)$$

$$\sigma_3 = \sqrt{\Delta t}\sqrt{\eta_1\frac{X_1^{n_1}X_2^{n_2}}{(X_1^{n_1} + K_1^{n_1})(X_2^{n_2} + K_2^{n_2})} + \eta_2\frac{K_1^{n_1}X_2^{n_2}}{(X_1^{n_1} + K_1^{n_1})(X_2^{n_2} + K_2^{n_2})}}$$
$$\sqrt{+\eta_3\frac{X_1^{n_1}K_2^{n_2}}{(X_1^{n_1} + K_1^{n_1})(X_2^{n_2} + K_2^{n_2})} + \eta_4\frac{K_1^{n_1}K_2^{n_2}}{(X_1^{n_1} + K_1^{n_1})(X_2^{n_2} + K_2^{n_2})} + \alpha_3 X_3 + r_3} \tag{14}$$

6.3 Expanded Results

Step 1. Fig. 9 shows the joint posterior distributions obtained in the 2nd inference step. Table 13 reports the posterior summaries for all parameters in Step 1. Presence of the activation/repression or both is inferred through θ and ω values. And the parameters inferred close to 0 are pruned.

Step 2. Using the inferred topology from Step 1, we perform the second inference step with the reduced parameter space. Table 14 summarizes the posterior distributions, showing tight credible intervals and accurate recovery of the true parameters.

6.4 Identifiability Considerations

To illustrate the identifiability issues that motivate the two-step inference procedure, Fig. 10 shows regions of parameter space where different combinations of interaction strengths yield nearly indistinguishable trajectories.

6.5 Kappa Values

We provide the posterior mean shrinkage factors κ under the regularized horseshoe prior, defined as $\kappa = \frac{1}{1+\tau^2\lambda^2}$. Values close to 1 indicate strong shrinkage toward zero, while values close to 0 indicate weak shrinkage (active parameters). We used 0.8 as the threshold for κ for pruning interactions. Table 13 summarizes the posterior mean values.

7 Identifiability

Several conditions can cause identifiability to fail, either structurally or practically:

Hill function saturation. Identifiability of regulatory interactions requires that the stationary distribution of the regulating TF X_j spans the transition region of the Hill function around K_j. When $X_j \gg K_j$ (saturated activation) or $X_j \ll K_j$ (saturated repression), the Hill function is approximately constant and insensitive to fluctuations in X_j. In this regime, the likelihood becomes flat with respect to both the interaction strength and type, and activation and repression produce indistinguishable transition densities. As we already noted in section 2.4 and Section 3.1 of the manuscript, and as illustrated in Fig. 1(e–f),a necessary practical condition for identifiability is therefore that the coefficient of variation of X_j at stationarity is non-negligible relative to K_j. This condition was enforced in all synthetic examples we presented.

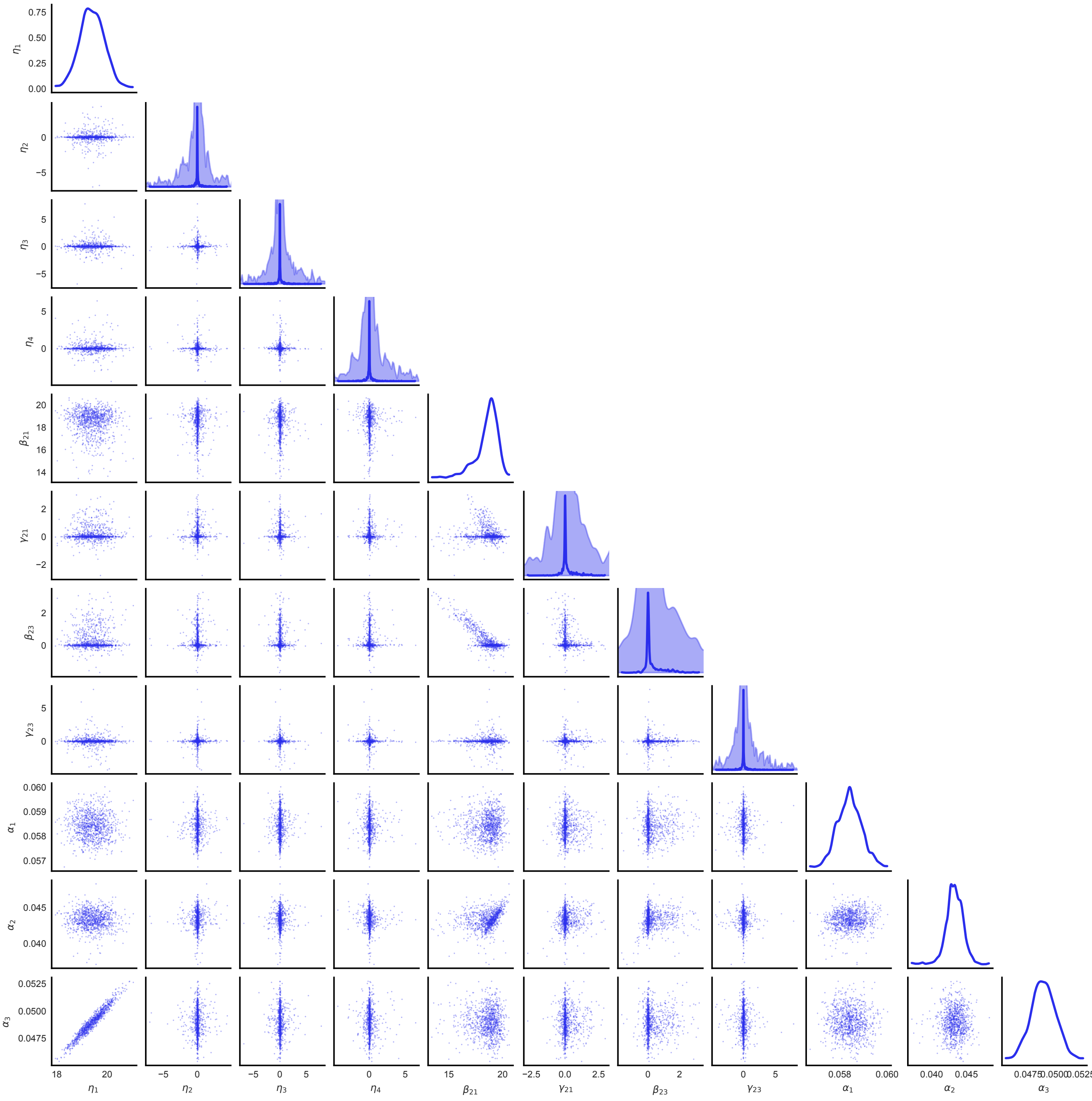

Figure 9: Joint posterior distributions obtained in the 2nd inference step

Table 13: Posterior summary statistics for Step 1 of the Coherent Type 1 motif.

Params	True	Mean	SD	Shrinkage (κ)	HDI 3%	HDI 97%	ESS Bulk	ESS Tail	$\hat{R}$
ρ_1	0	−0.042	0.370	0.928	−0.613	0.267	23 914.913	19 397.848	1.000
ρ_2	0	8.588	9.793	0.480	−0.609	20.960	213.942	2 114.809	1.013
ρ_3	20	5.271	8.055	0.600	−0.660	18.799	706.545	3 690.962	1.007
ν_{12}	0	−0.049	0.185	0.943	−0.414	0.177	22 701.251	14 995.177	1.000
ν_{13}	0	−0.028	0.360	0.939	−0.431	0.213	32 530.444	20 654.269	1.000
ν_{21}	20	11.215	9.794	0.368	−0.512	20.967	211.805	2 262.368	1.012
ν_{23}	0	−0.083	0.519	0.863	−1.356	0.742	18 479.432	14 897.431	1.000
ν_{31}	0	5.960	8.277	0.564	−0.544	18.734	718.821	3 988.731	1.006
ν_{32}	0	6.719	8.566	0.529	−0.507	18.951	583.013	4 012.485	1.012
θ_{12}	1	0.824	0.381	-	0.000	1.000	10 628.357	10 628.357	1.000
θ_{13}	1	0.790	0.407	-	0.000	1.000	8 326.047	8 326.047	1.001
θ_{21}	1	0.945	0.228	-	1.000	1.000	2 001.412	2 001.412	1.002
θ_{23}	1	0.854	0.353	-	0.000	1.000	9 656.289	9 656.289	1.000
θ_{31}	1	0.903	0.295	-	0.000	1.000	4 554.071	4 554.071	1.001
θ_{32}	1	0.908	0.290	-	0.000	1.000	4 544.547	4 544.547	1.002
ω_{12}	1	0.829	0.377	-	0.000	1.000	11 740.590	11 740.590	1.000
ω_{13}	1	0.808	0.393	-	0.000	1.000	9 376.912	9 376.912	1.000
ω_{21}	1	0.907	0.291	-	0.000	1.000	1 687.153	1 687.153	1.002
ω_{23}	1	0.906	0.292	-	0.000	1.000	1 639.116	1 639.116	1.002
ω_{31}	1	0.886	0.318	-	0.000	1.000	4 565.275	4 565.275	1.002
ω_{32}	1	0.884	0.320	-	0.000	1.000	4 548.106	4 548.106	1.001

Table 14: Posterior summary statistics for Step 2 inference.

Parameter	True	Mean	SD	HDI 3%	HDI 97%	ESS Bulk	ESS Tail	$\hat{R}$
η_1	20	19.395	0.497	18.488	20.357	33 939.951	16 720.494	1.00
η_2	0	0.003	0.725	−1.299	1.253	31 460.786	27 022.747	1.00
η_3	0	−0.007	0.730	−1.199	1.295	33 907.621	28 398.228	1.00
η_4	0	0.001	0.725	−1.287	1.182	32 878.182	28 048.499	1.00
β_{21}	20	18.528	1.124	16.204	20.215	29 751.600	32 140.395	1.00
γ_{21}	0	0.157	0.507	−0.560	1.409	24 974.224	26 209.454	1.00
β_{23}	0	0.296	0.637	−0.425	1.872	20 392.282	20 526.229	1.00
γ_{23}	0	0.005	0.740	−1.218	1.297	29 365.614	25 817.215	1.00
α_1	0.06	0.058	0.000	0.057	0.059	38 535.469	28 751.109	1.00
α_2	0.045	0.043	0.001	0.041	0.046	33 525.902	16 425.766	1.00
α_3	0.05	0.049	0.001	0.047	0.051	32 694.696	16 178.150	1.00

Linear dependencies in combinatorial regulation models. In the C1-FFL example, we observed strong posterior correlations between parameters governing AND-gate and single-input regulation at the same gene, for instance between ρ_2 and ν_{21}, and between ρ_3, ν_{31}, and ν_{32}. This reflects a structural identifiability issue. The Fisher information matrices for these parameter

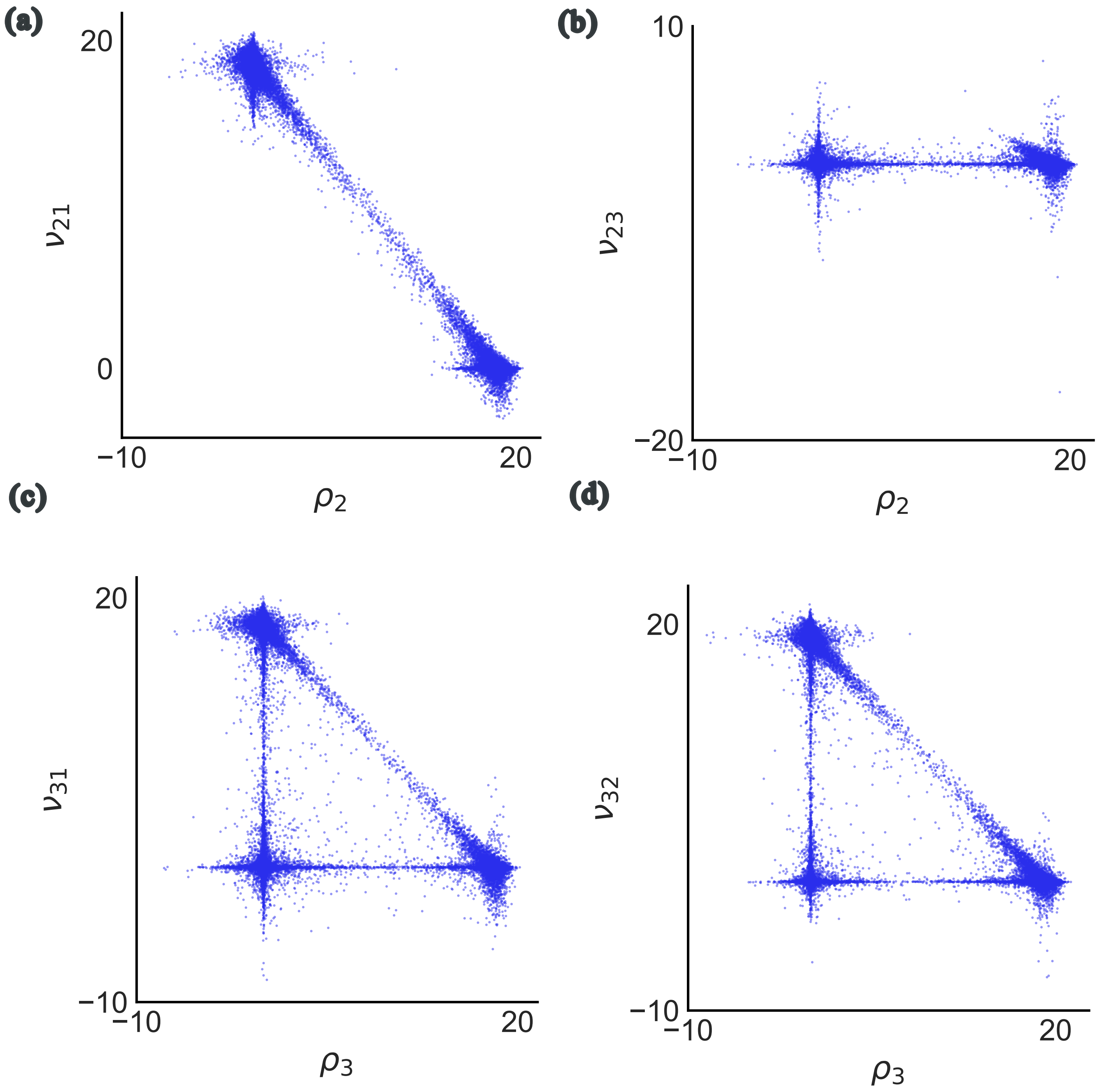

Figure 10: Illustration of identifiability issues for the Coherent Type 1 motif for step 1 of the inference procedure.

sets evaluated at the fixed points of the deterministic dynamics are nearly singular,

$$\mathcal{I}(\rho_2, \nu_{21}) \approx \begin{pmatrix} 1.661 & 1.656 \\ 1.656 & 1.661 \end{pmatrix}, \qquad \mathcal{I}(\rho_3, \nu_{31}, \nu_{32}) \approx \begin{pmatrix} 1.557 & 1.545 & 1.559 \\ 1.545 & 1.553 & 1.558 \\ 1.559 & 1.558 & 1.582 \end{pmatrix}, \tag{15}$$

confirming that these parameters are difficult to identify from stationary data alone. This motivated the two-step inference procedure, in which the network architecture is first identified before rate parameters are estimated. Correlation patterns

from the first inference step are reported in Fig. 10.

Network size. Inference for N genes involves $O(N^2)$ rate parameters, and as N grows the marginal variation of any single TF may be insufficient to identify its regulatory contributions. The increased number of horseshoe parameters further compounds issues of convergence and mixing in HMC sampling. Scaling to larger networks will require approximate inference or dimensionality reduction.

Beyond the weak identifiability of the Hill parameters K and n, the edge strength parameters β_{ij}, γ_{ij}, and η_{ij} are also subject to practical identifiability limitations depending on the operating regime of the Hill function. In the saturated regime $(X_j \gg K_j)$, the activation Hill function satisfies $f_a(X_j) \approx 1$, becoming insensitive to fluctuations in X_j, and making it difficult to attribute the drift contribution to a specific regulatory interaction. Conversely, $f_r(X_j) \approx 0$ in this regime, so the repression strength γ_{ij} becomes practically unidentifiable as its contribution to both $\mu(\boldsymbol{X})$ and $\sigma^2(\boldsymbol{X})$ vanishes. In the near-linear regime $(X_j \ll K_j)$, the Hill function behaves approximately as $f_a(X_j) \approx (X_j/K_j)^n$, so the drift depends on the product β_{ij}/K_j^n rather than on β_{ij} and K_j separately — a structural non-identifiability that cannot be resolved by additional data alone. Reliable inference of edge strengths therefore requires that the stationary distribution of X_j be contained in the transition region of the Hill function around K_j, a condition enforced in all synthetic examples in this paper and illustrated in Fig. 1(e–f). These limitations apply equally to the combinatorial regulation strengths η, since the AND-gate Hill functions in Eq. (2) are subject to the same regime-dependent approximations

8 Comparison with a Linearized VAR(1) Baseline

To situate our CLE-based inference relative to linearization-based approaches, we implemented a VAR(1) baseline by linearizing the CLE drift around the empirical steady state. The purpose of this comparison is to assess whether the full nonlinear CLE likelihood provides meaningful improvements over a simpler linear approximation, given the same prior structure and data.

Specifically, we linearized the drift of the full CLE model, which includes all potential regulatory interactions, just as in the nonlinear inference, around the empirical mean expression levels $(\bar{X}_1, \bar{X}_2)$. The Jacobian of the drift was computed analytically using Hill function derivatives:

$$J_{ij} = \left.\frac{\partial \mu_i}{\partial X_j}\right|_{\mathbf{X}^*}, \tag{16}$$

where μ_i denotes the drift of gene i as defined in the full inference model (e.g., Eq. (6) in the main text), which encompasses all possible activation and repression terms. The parameters $\boldsymbol{\beta}$, $\boldsymbol{\gamma}$, and $\boldsymbol{\alpha}$ entering the Jacobian are the same unknowns to be inferred; the linearization simply replaces the state-dependent Hill functions with their first-order Taylor expansions around $\mathbf{X}^*$, and approximates the diffusion as constant. The resulting linearized increment model is:

$$\Delta\mathbf{X} = \mathbf{J}(\boldsymbol{\beta}, \boldsymbol{\gamma}, \boldsymbol{\alpha})\,(\mathbf{X} - \mathbf{X}^*)\,\Delta t + \boldsymbol{\sigma}(\mathbf{X}^*;\, \boldsymbol{\beta}, \boldsymbol{\gamma}, \boldsymbol{\alpha})\,\sqrt{\Delta t}\,\boldsymbol{\epsilon}, \tag{17}$$

where $\boldsymbol{\epsilon} \sim \mathcal{N}(\mathbf{0}, \mathbf{I})$. This corresponds to the Euler–Maruyama discretization of an Ornstein–Uhlenbeck process centered at $\mathbf{X}^*$. Crucially, the network architecture is *not* assumed known: the Jacobian retains all potential interaction terms, and the same regularized horseshoe priors are applied to $\boldsymbol{\beta}$ and $\boldsymbol{\gamma}$ as in the nonlinear CLE model. The horseshoe prior is responsible for shrinking absent interactions toward zero in both cases. The only difference between the two approaches is that the VAR(1) model uses a linear approximation to the drift and a constant diffusion, whereas the CLE model retains the full nonlinear, state-dependent drift and diffusion.

Note: The generating parameters for the two-gene VAR(1) comparison ($\gamma_{21} = 25$, $\alpha_1 = 0.01$, $\alpha_2 = 0.03$) differ from those used in the main text toggle switch example ($\gamma_{21} = 20$, $\alpha_1 = 0.03$, $\alpha_2 = 0.02$); this separate parameterization was chosen to test both methods under stronger repressive regulation.

Both methods correctly shrink absent interactions toward zero. However, three differences are notable. First, the CLE yields approximately three times tighter posterior uncertainty on β_{11} in the one-gene case (std 0.40 vs 1.28), reflecting the additional information captured by the state-dependent diffusion term. Second, VAR underestimates the strong repression parameter γ_{21} by ~24% (mean 19.10 vs true 25), whereas CLE recovers it with ~4% error (mean 23.95). This systematic

Table 15: Posterior mean ± std for CLE and VAR(1) on the one-gene and two-gene motifs. Both methods used identical horseshoe priors. True parameter values are given for reference.

Parameter	True	CLE mean ± std	VAR mean ± std
One-gene motif			
β_{11} (activation)	10.0	9.09 ± 0.40	9.01 ± 1.28
γ_{11} (repression, absent)	0.0	0.001 ± 0.50	−0.014 ± 0.47
α_1 (degradation)	0.05	0.052 ± 0.006	0.052 ± 0.006
Two-gene toggle switch			
β_{12} (activation, $1 \to 2$)	10.0	9.60 ± 0.33	9.77 ± 0.90
β_{21} (activation, absent)	0.0	−0.070 ± 0.230	−0.074 ± 0.341
γ_{21} (repression, $2 \to 1$)	25.0	23.95 ± 5.37	19.10 ± 7.97
γ_{12} (repression, absent)	0.0	0.100 ± 0.239	0.057 ± 0.294
α_1 (degradation)	0.01	0.009 ± 0.001	0.010 ± 0.001
α_2 (degradation)	0.03	0.028 ± 0.002	0.037 ± 0.007

bias arises because the Hill function is evaluated far from its inflection point, where the local linear approximation poorly captures the true regulatory sensitivity. Third, degradation rate estimation is comparable in the one-gene case but CLE provides substantially tighter estimates in the two-gene case (α_2 std 0.002 vs 0.007). Together, these results suggest that VAR/OU approximations are adequate for weak interactions near the linear Hill function regime, but the full nonlinear CLE is preferable when strong regulatory interactions must be accurately quantified.

9 Effect of Sampling Interval and Trajectory Length on Inference

9.1 Sampling Interval (Δt)

We investigated the sensitivity of inference to the choice of sampling interval Δt using the two-gene toggle-switch motif. Too small a Δt yields high computational cost, as many time steps are needed to accumulate sufficient evidence, and can also lead to larger posterior variance due to high correlation between consecutive observations. Too large a Δt causes the Euler–Maruyama discretization to break down, leading to sampler divergences and degraded inference. A moderate Δt, chosen so that consecutive observations are approximately uncorrelated while maintaining a faithful discretization of the CLE, provides the best balance of accuracy and efficiency. Fig. 11 illustrates this trade-off.

9.2 Trajectory Length

We examined the effect of trajectory length on inference quality using the two-gene toggle-switch motif (Fig. 12). The effect is parameter-specific: with $L = 100$ observations, the activation parameter β_{12} is recovered reasonably, but the repression parameter γ_{21} (true value = 20) is confidently misidentified as zero, representing complete inference failure rather than merely high uncertainty. As trajectory length increases, posterior variance decreases monotonically and inference improves, with reliable recovery of all parameters at $L \geq 500$. The required trajectory length is expected to grow with network size due to the $O(N^2)$ scaling of parameters.

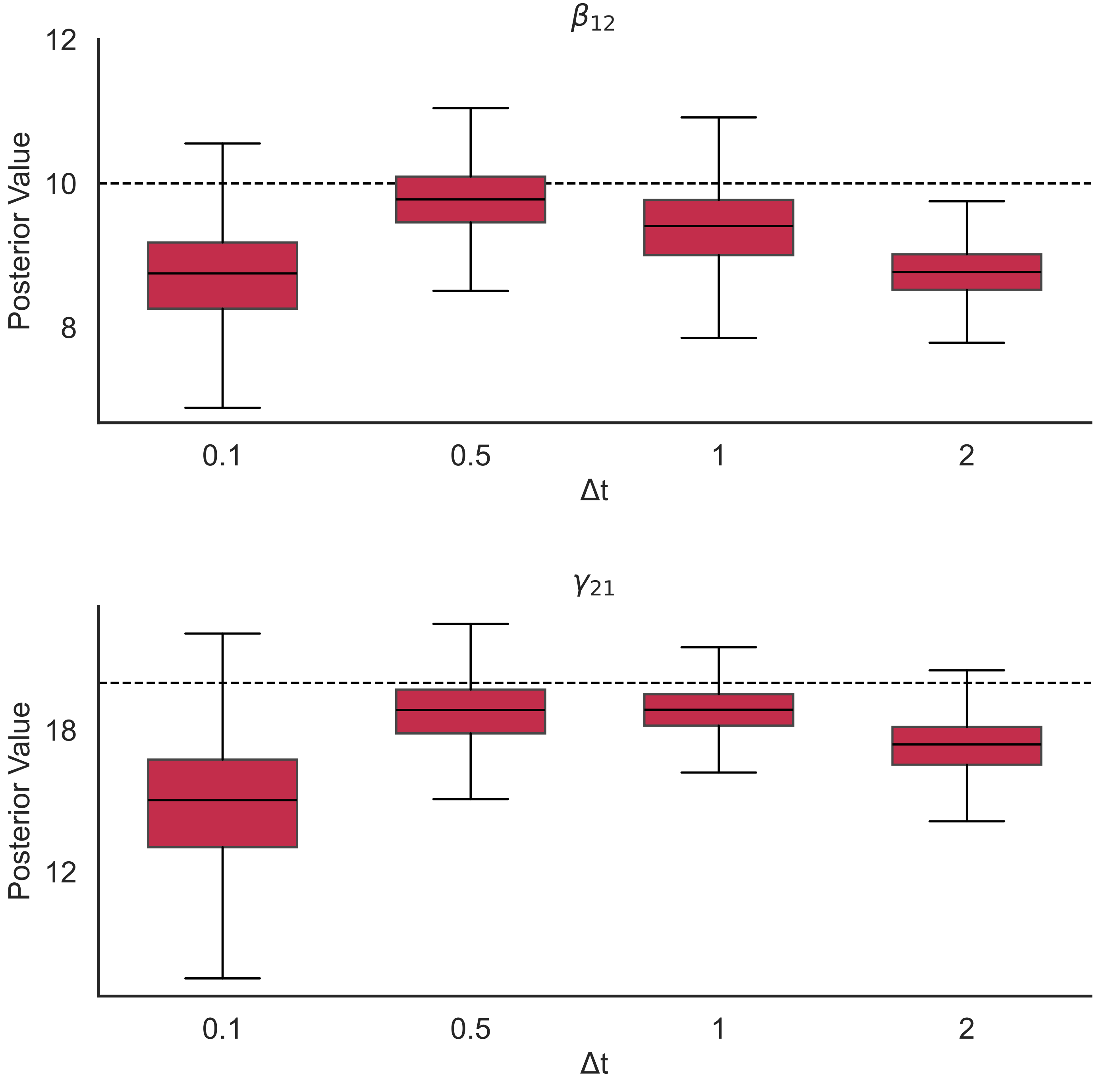

Figure 11: Effect of sampling interval Δt on inference in the two-gene motif. Boxplots of posterior means of the activation parameter β_{12} (top, true value = 10) and repression parameter γ_{21} (bottom, true value = 20) across $\Delta t \in \{0.1, 0.5, 1, 2\}$. At small $\Delta t = 0.1$, posterior variance is large due to high temporal correlation between consecutive observations. Intermediate values ($\Delta t = 0.5$–1) yield the most accurate estimates with tightest interquartile ranges. At $\Delta t = 2$, estimates begin to degrade as the Euler–Maruyama discretization becomes less faithful. Dashed lines indicate true parameter values.

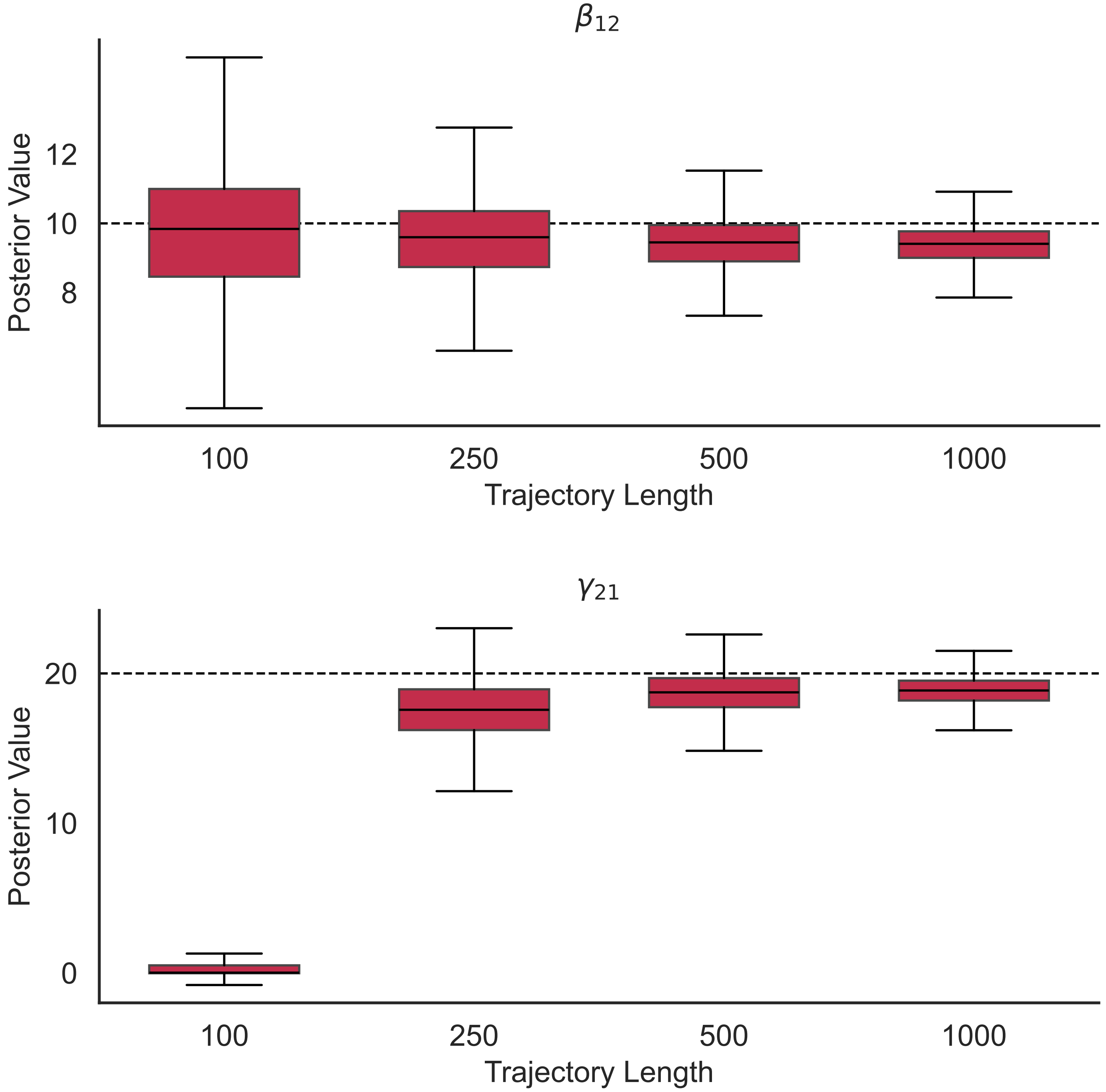

Figure 12: Effect of trajectory length on inference in the two-gene motif. Boxplots of posterior means of the activation parameter β_{12} (top) and repression parameter γ_{21} (bottom) across increasing trajectory lengths. Posterior variance decreases with longer trajectories, indicating improved practical identifiability. Dashed lines indicate true parameter values.
